# Quantitative Modeling of Polaritonic Emission Using the Source Term Method

**DOI:** 10.1021/acs.jpclett.5c01213

**Published:** 2025-06-17

**Authors:** Rahul Bhuyan, Maksim Lednev, Clara Schäfer, Johannes Feist, Karl Börjesson

**Affiliations:** † Department of Chemistry and Molecular Biology, University of Gothenburg, Gothenburg 41390, Sweden; ‡ Departamento de Física Teórica de la Materia Condensada and Condensed Matter Physics Center (IFIMAC), Universidad Autónoma de Madrid, Madrid 28049, Spain

## Abstract

Strong exciton-photon coupling leads to the formation of hybrid states, polaritons, with properties different from those of their constituents, making it a valuable tool for modifying the physical and chemical properties of organic and inorganic materials. Despite its potential, the field lacks a fundamental understanding of the photophysics involved and the ability to model experimental data effectively. In this study, we quantitatively simulate polaritonic emission using the source term method. This model assumes that each molecular dipole in the exciton reservoir emits as it would in free space, into the optical environment formed by the polaritons. To benchmark theory with experiments, a BODIPY derivative containing a suitable amount of steric bulk was synthesized. Neat films of this molecule exhibited close to unperturbed absorption and emission envelopes compared to dilute solution. When placed in an optical cavity, the ultrastrong coupling regime was reached, and a collapse of the polaritonic line width was observed. Such a collapse is an indication of an ideal polariton, and it allowed for the emission in the transverse electric and magnetic polarizations to be spectrally resolved and thus successfully compared to the simulated emission. This work hence describes an effective model that fits experimental data, which is crucial for advancing the field and for optimizing applications.

## Graphical abstract

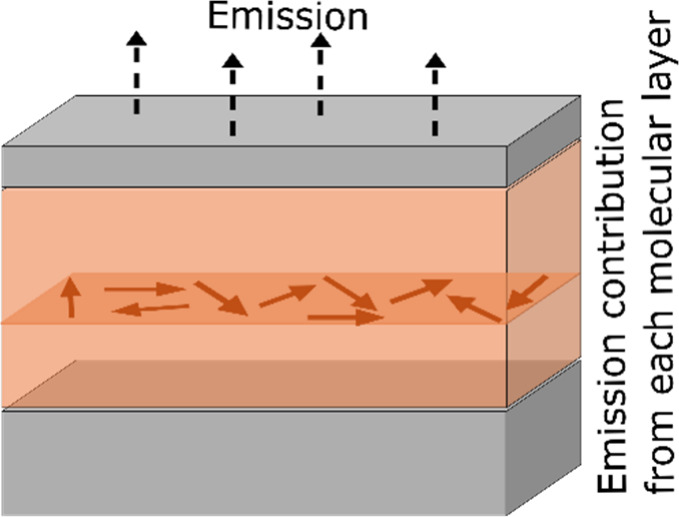

Strong exciton-photon coupling enables the manipulation of the physical and chemical properties of both organic and inorganic materials.^1–7^ The research area has gained significant attention in recent years for its wide-ranging potential applications, such as modified chemical reactivity,^8–11^ enhanced reversed intersystem crossing,^12–14^ facilitated long-distance energy transfer,^15–20^ influenced rate of singlet fission and triplet–triplet annihilation,^21–25^ enhanced organic electronics,^26–32^ and even the ability to form room temperature Bose–Einstein condensation.^33,34^ Despite the demonstrated potential for broad applications, there is still a lack in the understanding of the underlying photophysics and in the ability to model experimental data accurately. There is therefore a need to further develop models that correctly explain experimental data in order to fine-tune systems for the desired photophysics and the optimal performance.

The strong interaction of many degenerate molecular transitions with the photonic modes of an optical cavity in ideal circumstances leads to the formation of two polaritonic modes that gain all photonic contribution, along with a reservoir of many degenerate “dark” states that are superpositions of molecular excitations that do not couple to the cavity modes.^35–37^ These polaritonic states, known as the lower and upper polariton, are separated in energy by the Rabi splitting at their point of nearest approach, and exhibit an angular dispersion in energy. They form a new optical environment for the molecules. The angular dispersion of the upper and lower polaritons is often modeled using a coupled harmonic oscillator model,^38^ while the optical environment is often modeled using the transfer matrix method.^39^ When the photonic modes are created by a Fabry–Perot cavity, the cavity energy dispersion differs slightly between the transverse electric (TE, where the electric field is normal to the plane of the incident light) and the transverse magnetic (TM, where the electric field is within the plane of the incident light) polarizations. As a result, the polaritonic dispersion in the TE and TM polarizations also differs. Studies have shown spectrally resolved polaritonic reflectivity and emission;^40–44^ however, these emission intensities have rarely been quantified. Quantifying the contribution of the spectrally resolved emissions for the TE and TM polarizations is a necessary step toward developing more accurate theories on excited state processes in polaritonic systems.

To measure the TE and TM emission of the lower polariton quantitatively, a system with spectrally resolved TE and TM emissions is required, which requires smaller polariton line widths than achieved with most organic molecules due to their significant inhomogeneous broadening. When the Rabi splitting significantly exceeds the inhomogeneous broadening of the molecular transition, the line widths of the formed polaritons are reduced and approach the average of the molecule’s natural line width and that of the cavity.^45^ This line width collapse is indicative of an “ideal” polariton as mentioned above, for which only the upper and lower polaritons exhibit photonic contributions from the cavity mode. Organic molecules with a high transition dipole moment in combination with a narrow line width in the solid state are excellent candidates to achieve such line width collapse, as has for instance been shown using the molecule squaraine.^46^ Furthermore, to experimentally observe spectrally resolved TE and TM emission, a significant solid-state emission quantum yield of the molecule is needed.

In this study, we experimentally form an ideal polariton using a synthesized BODIPY derivative. A collapse of the polariton line width is observed, which serves as an indicator of the idealness of the lower polariton. This collapse allows for the quantitative collection of spectrally resolved TE and TM emission from the lower polariton. We further calculated the lower polaritonic emission for both the TE and TM polarizations using the source term method,^47^ which assumes that emission from the dark-state reservoir proceeds with the free-space emission spectrum of the molecules filtered by the polaritons. The calculated emission matches well with the experimental polaritonic emission in terms of energy and intensity of the TE and TM dispersion. In comparison, the widely adopted spectral multiplication method does not show as good match with experimental data. These findings suggest that the polaritonic emission is the result of emission of molecules in free space filtered through the modified optical environment provided in the strong coupling regime.

Fabry–Perot cavities were used to provide an optical mode strongly coupled to the optical transitions of a layer of organic molecules. These cavities contain two parallel mirrors sandwiching an organic layer, the thickness of which dictates the cavity resonance energy. To increase emission outcoupling, cavities had unsymmetric mirror thicknesses. Cavities were fabricated on a clean glass surface by initially sputtering a thick Ag layer (100 nm). Subsequently, a pristine molecular layer was spin-coated on top. Finally, a semitransparent Ag layer (30 nm) was deposited (Figure S1). Such structure resulted in cavities having a quality factor of around 60 (Figure S2). The thickness of the molecular layer was adjusted to prepare cavities with different energy detuning relative to the excitonic energy, and this study presents data from three such cavities (see Supplementary section 1 for details). To achieve the strong coupling regime and spectrally resolved TE and TM emission, an organic dye with a high transition dipole moment and narrow absorption line width is necessary. To fulfill these requirements, we synthesized a new BODIPY derivative for this study (Figure 1a). In the design of the molecule, the π-conjugated scaffold was alkylated using bulky substituents to reduce intermolecular π-π interactions, thereby minimizing the formation of ground-state or excited-state trap states in the solid state. The synthesis methodology follows previous reports,^48,49^ and the detailed synthetic procedure and chemical characterization are presented in the Supplementary section 2.

**Figure 1:**
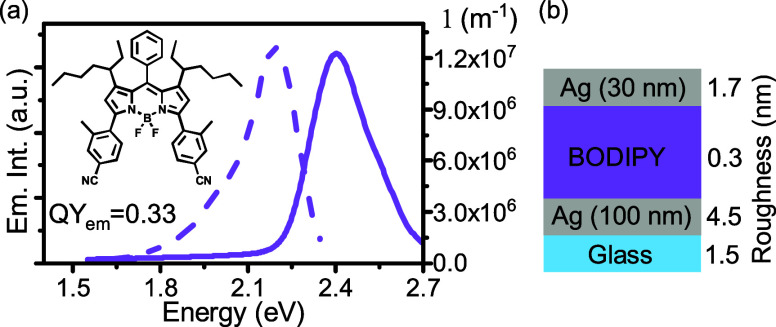
(a) Solid-state absorption (solid line) and emission (dotted line) spectra of a neat film of the BODIPY derivative; along with its molecular structure. (b) Cavity structure from top to bottom: Ag (30 nm), BODIPY neat film, Ag (100 nm), glass substrate. The mean square roughness of the surface after each deposition is given next to each layer.

Figure 1a shows the solid-state absorption (solid line) and emission spectra (dotted line) of the molecule. The absorption spectrum displays a main transition at 2.403 eV and a smaller hump at 2.524 eV. The envelope of absorption is slightly broader in the solid state compared to in the solution (Figure S3), and the overall line width is 222 meV, which is relatively small.

The emission spectrum is a mirror image of the absorption, and it is slightly red-shifted when going from solution to the solid state (Figure 1 and S4). It features a main transition at 2.196 eV and a smaller hump at 2.02 eV. The emission quantum yield is 0.33 ± 0.02 directly after spin coating, which is unusually high for films of pristine organic dyes. A detailed description of the photophysical properties of the BODIPY derivative is given in the Supporting Information (Figures S5 and S6 and Table S1).

To confirm that the strong coupling regime was entered, the angle-dependent reflectivity of cavities was measured in both the transverse electric (TE) (as shown in Figure 2) and transverse magnetic (TM) polarizations (as shown in Figure S7). The minima of the angle-dependent reflectivity spectra were then fitted using the coupled harmonic oscillator model

1 $$E_{UP/LP}\left(k_{∥}\right)=\frac{1}{2}\left(E_{X}+E_{C}\left(k_{∥}\right)\right)\pm \sqrt{V_{a}^{2}+\frac{1}{4}{\left(E_{X}-E_{C}\left(k_{∥}\right)\right)}^{2}}$$

where *E*_UP_ and *E*_LP_ are the energies of the upper and lower polaritons, respectively. *E*_*x*_ is the energy of the electronic transition being coupled (2.43 eV), which was determined as the weighted average of the two vibronic transitions in the BODIPY absorption spectrum (Supplementary section 3.1 and Figure S8). *E*_c_ is the cavity energy, which depends on the in-plane momentum (*k*_∥_) and the cavity mode order (*m*). The energy of the *m*th order cavity mode as a function of in-plane momentum is given by

$$E_{C}\left(k_{∥}\right)=\frac{\hbar c}{n_{eff}}\sqrt{k_{∥}^{2}+{\left(\frac{m\pi }{L_{cav}}\right)}^{2}}$$ 2

3 $$k_{∥}=\frac{2\pi }{\lambda }\sin\theta$$

where *c* is the speed of light, θ and λ are the angle and wavelength of the incident light, respectively, and *n*_eff_ and *L*_cav_ are the effective refractive index and thickness of the cavity, respectively. In the TE polarization, fits for the lower and upper polaritons were performed for all the cavities, keeping *n*_eff_ as a global parameter, and *L*_cav_ and *V*_a_ as individual parameters. The cavity energies at *k*_∥_ = 0 (*E*_C_(0)) could in this manner be extracted from the reflectivity spectra.

**Figure 2:**
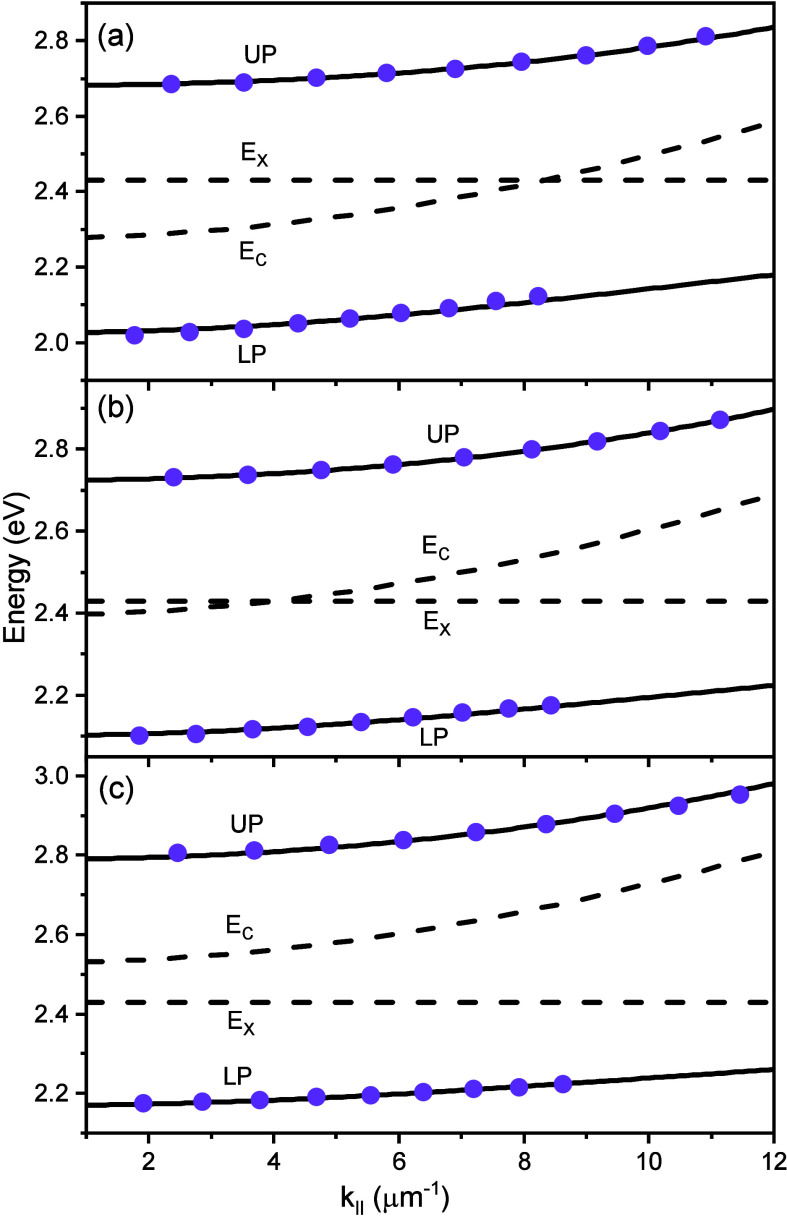
Energy of the upper and lower polaritons (taken as the reflectivity minima) as a function of in-plane momentum for the transverse electric (TE) polarization. Here, the molecular transition was coupled to cavities having a λ/2 mode with *E*_c_(0) at (a) 2.277 eV, (b) 2.395 eV, and (c) 2.529 eV. The solid circles correspond to the experimental polaritonic energies, the solid lines represent the fit of the polaritonic dispersion using the coupled harmonic oscillator model, and the dotted lines correspond to the energies of the molecular transition and cavities.

The electromagnetic field distribution differs for the TE and TM polarizations in a microcavity. The effective cavity length and effective refractive index are therefore different for the TM modes as compared to the TE ones. Furthermore, in the TM polarization, the effective refractive index depends upon *L*_cav_.^40,50^ To compensate for these effects in the TM polarization, fits were performed individually for each cavity, using the *E*_C_(0) values attained from the TE polarization fits, and *V*_a_, *L*_cav_, and *n*_eff_ as free parameters. All the fitting parameters for both the TE and TM polarizations are given in Table S2.

The *V*_a_ of the cavities vary from 306 to 319 meV for the TE polarization and from 304 to 321 meV for the TM polarization. The *V*_a_ is thus significantly larger than the average of the full width half-maximum of the molecular transition (222 meV) and the cavity mode (42 meV). This observation indicates that the system is in the strong coupling regime for both the TE and TM polarizations.^51^ Furthermore, the coupling strength is comparable to the excitonic transition energy (*V*_a_/*E*_*x*_ > 12.5%), placing the system in the ultrastrong coupling regime.

The angle-resolved polaritonic emission was measured for both the TE and TM polarizations to assess their relative contributions. Figure 3 (and Figure S9) shows the isotropic, the TE and the TM emissions. Notably, a split in the lower polaritonic emission is observed at higher angles for the isotropic case. This split is more pronounced for negatively detuned cavities (*E*_*C*_(0) < *E*_*X*_). The energy of the lower polariton, as measured by reflectivity, is overlaid on the emission contour plots. The energy of the lower polariton in the TE and TM polarizations precisely matches with the two maxima seen in the isotropic emission. Furthermore, when the emission was measured in the TE and TM polarizations, the single emission maxima observed matched the respective polariton energies (as measured with reflectivity). Thus, the two maxima observed in the isotropic emission are clearly due to the TE and TM polaritons, and they can be spectrally resolved.

**Figure 3:**
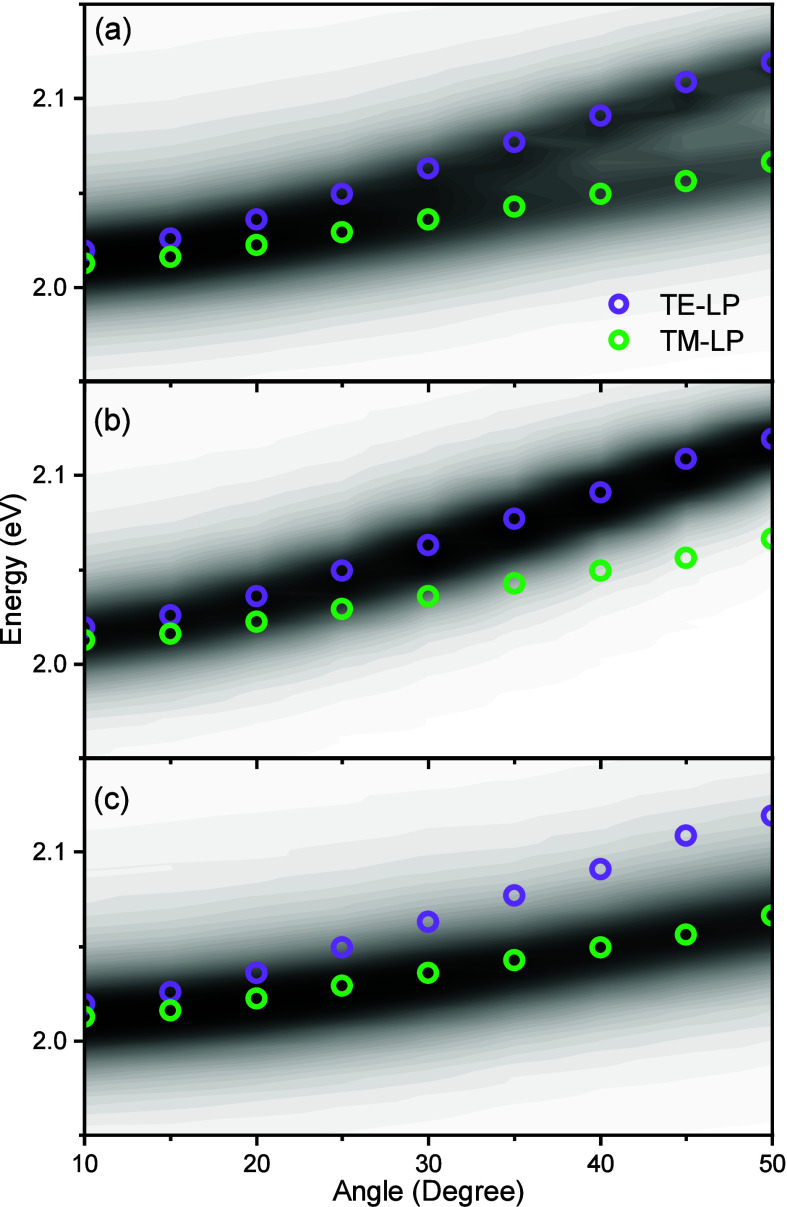
Angle-dependent emission contour plots for the cavity having a *E*_c_(0) = 2.277 eV. The excitation energy was at 2.403 eV (thus nonresonantly) and was performed at an angle of 15°. To avoid reflection into the detector, the emission was captured orthogonal to the plane of excitation. The purple and green circles correspond to the reflectivity minima of the lower polariton in the transverse electric (TE) and transverse magnetic (TM) polarizations, respectively. The emission spectra were recorded (a) isotropically, (b) in TE polarization, and (c) in TM polarization. In the plots, white indicate low emission and black indicated maximum emission intensity.

The experimental observation of the spectrally resolved energies of the TE and TM polaritons is an implication of their narrow line widths. In our system, the Rabi splitting (608–642 meV) is significantly larger compared to the inhomogeneous broadening of the molecular transition (∼222 meV). For such cases it has been shown theoretically that the polariton line width approach that of the average of the homogeneous line width of the molecule and the cavity mode.^45^ The homogeneous line width of the molecular transition was approximated by fitting the absorption spectrum with a Voigt function (Supplementary section 3.1 and Figure S8).^52,53^ The width of the Lorentzian part in the function was taken as the homogeneous line width (34–42 meV). The line widths of the lower polariton in the three cavities (30–50 meV; Figure S10) were on the same order of magnitude as the average of the line widths of the homogeneously broadened molecular transition and the cavity mode. This phenomenon has been observed before in the ultrastrong coupling regime,^40,46,54^ and can be viewed as an indication that the polaritons approach the ideal situation described above. In our case, the Rabi splitting is more than three times the inhomogeneous broadening of the molecular film. It has recently been suggested that such high splitting is necessary in order to achieve a delocalized polariton.^55^

For an ideal polaritonic system, only the upper and lower polaritons exhibit a photonic contribution from the cavity mode. To verify that the polaritons in our system are close to ideal, we calculated the photonic contribution to all hybrid states for a model system. It consisted of 1000 disordered emitters, with an energy distribution chosen to reproduce the experimental molecular absorption spectrum, and one cavity mode.^56^ For the coupling parameters reproducing the experimental Rabi splitting, we indeed observed that the states between the polaritonic bands are “dark”, i.e., show negligible photon contributions (Figure S11). The system is thus close to ideal, explaining the narrow line width of the lower polariton.

While polaritonic emission has been modeled in different contexts,^57–62^ quantitative studies comparing the lower polariton emission for both TE and TM polarizations have not been reported so far. So far, two mechanisms have been proposed to explain the emission from the lower polariton: 1) vibration-assisted scattering and 2) radiative pumping, which are two different but closely related relaxation pathways.^7,63^ Vibration-assisted scattering is mainly observed for molecules having small Stokes shifts, such as J-aggregates. However, for the case of molecules with large Stokes shifts, radiative pumping is the dominant mechanism for populating the lower polariton.^63^ Since our BODIPY derivative has a large Stokes shift, vibration-assisted scattering was ignored, and radiative pumping was considered as the main pathway of relaxation. Radiative pumping can be understood as the emission from “dark” reservoir states, which are essentially bare molecular states, into the optical environment formed by the polaritonic states.

In order to simulate the emission from the microcavities, we used the source term method combined with the transfer matrix method (TMM).^47^ As a first step, we fitted the reflectivity of all three cavities, which gives us full information about the thicknesses and dielectric properties of the layers (Figures S12–S15). Then, the emission from the microcavity was obtained by calculating the electric field distribution of the illumination within the cavity and treating the molecules in the active organic layer as point dipoles with random orientation, which were excited with different probabilities depending on the intensity of the excitation field at their position. We then used the source term method, with the assumption that each molecule emits according to its free-space emission spectrum, to calculate the emitted electric field outside the cavity. The angle-resolved intensity spectrum can then be obtained from

$$I^{pol}\left(\theta ,\omega \right)\propto I_{mol}\left(\omega \right)T_{t}\left(\theta ,\omega \right)=I_{mol}\left(\omega \right)\underset{i=x,y,z}{\sum }D_{i}^{pol}L_{i}\int_{\begin{array}{l} mol\\ layer \end{array}}dzA\left(z\right)P_{i}\left(\theta ,\omega ,z\right)$$ 4

where *I*_*mol*_ is the emission spectrum of the bare molecules, *T*_*t*_ is the effective transmission of the signal from within the cavity to outside, $P_{i}={\left|E_{i}\left(\theta ,\omega ,z\right)\right|}^{2}\cdot \frac{\cos^{2}\theta }{\cos^{2}\theta_{mol}}$ is the power emitted into solid angle θ (up to a prefactor which is independent of the frequency, angle and position), *E*_*i*_(θ,ω,*z*) is the electric field amplitude obtained through the TMM using the source method, θ_*mol*_ is the angle of light propagation in the molecular layer, *A*(*z*) is the position-dependent absorption of the excitation light, *L*_*i*_ represents the factor accounting for different probabilities of excitation for the dipoles with different orientation, and *D*_*i*_^*pol*^ is the projection of the emitted field polarization onto the axis of the detection polarizer (see Supplementary section 3.2 for details).

The simulated angle-resolved emission spectra for both polarizations together with the experimental data are shown in Figure 4 and Figures S16–S19. The dispersion of the energy maxima of the polaritonic emission is accurately captured by the simulations. Furthermore, the spectral envelope of the emission is also extracted well. This includes the fwhm, which equals 36 and 35 meV for the experimental and simulated widths, respectively (at 10 degrees for the cavity with energy 2.277 eV; the values for the TM mode are 35 and 37 meV).

**Figure 4:**
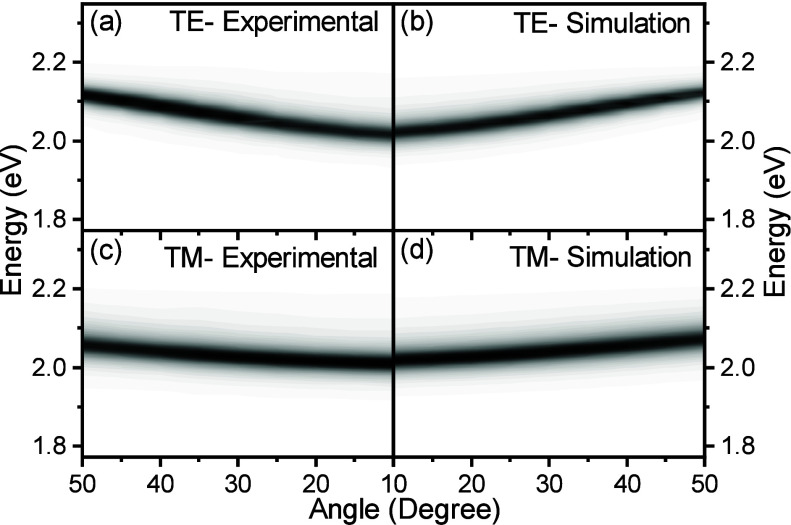
Emission intensity from the cavity having an energy of 2.277 eV measured in the (a) TE and (c) TM polarizations. Simulated emission from the cavity in the (b) TE and (d) TM polarizations. In the plots, white indicate low emission and black indicated maximum emission intensity.

To visualize the angular dependence of the emission intensity, the simulated and measured data were integrated and normalized for comparison (Figure 5, black and purple lines). Our theoretical model also captures the change in integrated emission intensity as a function of detection angle accurately. For all the cavities and both polarizations, the model results follow the distinctive experimental trends. We note that the good agreement between experiment and theory implies that surface roughness is small enough not to significantly affect the outcoupling, and in particular to not introduce significant loss channels such as scattering to surface plasmon polaritons. This is corroborated by the fact that all surfaces were smooth when examined by AFM (Figure S20).

**Figure 5:**
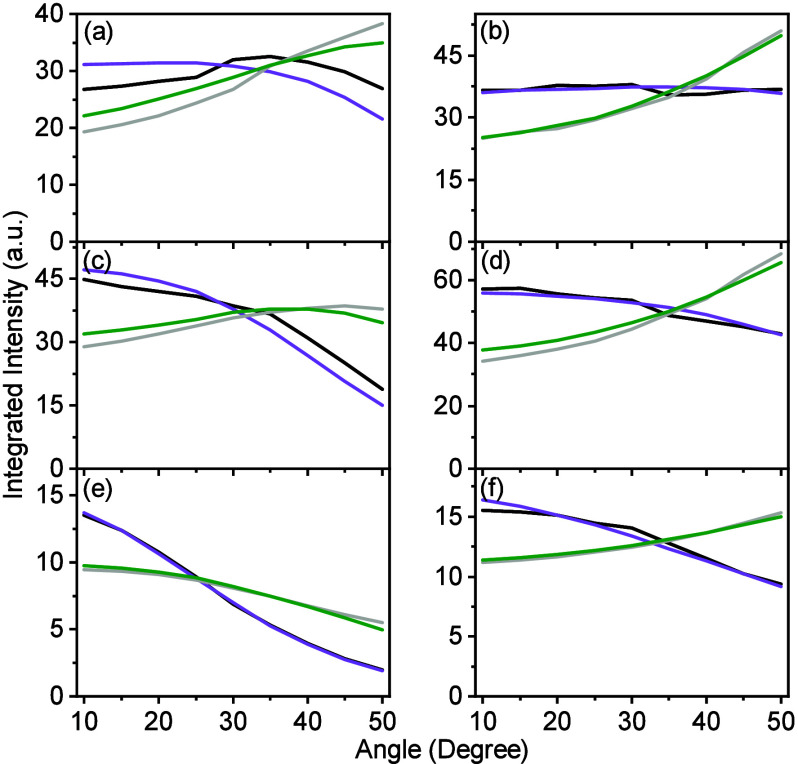
Integrated emission intensity from the three cavities measured in (a, c, e) the TE and (b, d, f) TM polarizations. Different rows correspond to samples with different cavity energies: (a, b) 2.277 eV, (c, d) 2.395 eV, and (e, f) 2.529 eV. The black (experimental emission), purple (Simulated emission), gray (spectral multiplication of the molecular emission and 1-R, where R is the experimental reflectivity of the cavity), and green (spectral multiplication of the molecular emission and 1-R, where R is the TMM calculated reflectivity of the cavity).

For comparison, simulating the system with the often-used simple model of emission reabsorption (i.e., multiplying the bare molecule emission with the polaritonic absorption spectra),^64^ the match between simulation and experiment fails. This applies both when using the experimental and TMM simulated reflectivity (gray and green lines in Figure 5, respectively). The reason why our method captures the experimental data well is because it considers the distribution of exciton density in the cavity, as well as the propagation of emitted light from the different parts of the active layer, correctly including all the optical properties and reflections (Figures S21 and S22). Each infinitesimal layer in the cavity therefore contributes differently to the overall emission from the cavity. Thus, to obtain an accurate simulation of the overall angular dependent emission, each layer needs to be individually considered, and their effect summed.

Here, we quantitatively measure and model the angular dependence of the polarization-resolved polaritonic emission. To do this, a BODIPY derivative was first synthesized, exhibiting narrow absorption lines in highly absorbing pristine films. Three Fabry–Perot cavities filled with the BODIPY derivative were fabricated, varying the cavity energy. The cavity modes strongly coupled with the S_0_→S_1_ transition of the BODIPY derivative, resulting in the formation of polaritonic states. These states were shown to be ideal, with only two hybrid states having a photonic character. Due to the ideal nature of the polaritons their line width collapsed, approaching the homogeneous broadening limit, allowing spectrally resolved TE and TM emission.

The TE and TM polarized emissions were simulated using the source term method, under the assumption that molecules in the exciton reservoir emit as they would in free space, into the optical environment formed by the polaritonic state. Our simulations successfully reproduce both the energy dispersion and the intensity of the angular resolved polaritonic emission. In contrary, the commonly used macroscopic model that uses the product of the polariton absorption and molecular emission failed to reproduce the angular dependence of the emission intensity. This suggests that the polaritonic emission can be modeled as the free-space emission of molecules in the exciton reservoir, filtered through the optical environment created by strong light-matter interactions. However, as we show here, the nonuniform density of excitons in a cavity needs to be taken into account in order for such simulations to be quantitatively accurate.

## Supplementary Material



## Data Availability

Raw data are stored at the Swedish National Data Service with the digital object identifier 10.5878/jh1e-7m58 (10.5878/jh1e-7m58).

## Notes

Additional experimental details, analyses, materials and methods, along with ^1^H and ^13^C NMR spectra and additional spectroscopic and simulated data (PDF)
