# Supplementary material for: Quantitative Modeling of Polaritonic Emission Using the Source Term Method
Source: J Phys Chem Lett. 2025 Jun 17;16(25):6435–41. doi: 10.1021/acs.jpclett.5c01213 (PMC12207670; doi:10.1021/acs.jpclett.5c01213)
Supplement: Supplementary file 1 [file jz5c01213_si_001.pdf]

Supplementary Information for

Quantitative Modeling of Polaritonic Emission Using the Source Term Method

Rahul Bhuyan,[a,§] Maksim Lednev,[b,§] Clara Schäfer,[a] Johannes Feist,[b,*] and Karl Börjesson[a,*]

[a]Department of Chemistry and Molecular Biology, University of Gothenburg, 41390 Gothenburg (Sweden).
[b]Departamento de Física Teórica de la Materia Condensada and Condensed Matter Physics Center (IFIMAC), Universidad Autónoma de Madrid, Madrid, E-28049 (Spain).

§ R.B. and M.L. contributed equally to this paper.
*Corresponding Author: karl.borjesson@gu.se and johannes.feist@uam.es

Table of Contents

1. Experimental Methods 3

1.1 Cavity Preparation 3

1.2 Steady State Absorption, Reflection, and Emission Spectroscopy 3

1.3 AFM Analysis 3

2. Synthesis of the BODIPY Derivative 4

3. Supplementary Analysis 8

3.1 Excitonic Energy and Homogenous Broadening Calculations 8

3.2 Emission Modelling 8

4. Supplementary Figures 10

5. ^1H and ^{13}C NMR 24

6. Supplementary Tables 29

7. Reference 30

1. Experimental Methods

1.1 Cavity Preparation

2.5 cm×2.5 cm glass substrates (microscope slides purchased from J. Melvin Freed) underwent a 15-minute sonication in a 1% alkaline solution (Hellmanex in Milli-Q water). Subsequently, they were sonicated for 1 hour in Milli-Q water and then ethanol. After the cleaning process, the glass substrates were dried in an oven overnight before the fabrication of films or cavities. The Ag mirrors were produced through vacuum sputtering deposition (HEX from Korvus Technologies). Initially, a 100 nm Ag mirror was sputtered onto the glass substrate. Subsequently, 100 μl of a solution of the BODIPY derivative (36 mg/ml in toluene) was spin-coated at 1400 to 2600 rpm on the surface of the Ag mirror. To complete the optical cavity, a semi-transparent 30 nm Ag mirror was sputtered onto the molecular film. The reflectivity and transmittance spectra of a 30 nm Ag mirror is shown in Figure S1.

1.2 Steady State Absorption, Reflection, and Emission Spectroscopy

Steady-state absorption of films and reflectivity of cavities were recorded using a Perkin Elmer LAMBDA 950 spectrometer. Angle-dependent reflectivity of cavities was measured by a Universal Reflectance Accessory (URA, Perkin Elmer). Spectra were referenced to a standard reflectance mirror inside the URA, employing a Glan-Taylor polarizer. Reflectivity spectra were collected in both transverse electric (TE), and transverse magnetic (TM) polarizations.

Steady-state emission spectra of the films and cavities were measured on an Edinburgh Instruments FLS 1000 spectrofluorometer, with a Xenon lamp as the excitation source and a double monochromator for wavelength selection (3 nm slit sizes for both excitation and emission). Angle-resolved emission spectra of the cavities were measured with isotropic, TE, and TM polarizations on the same instrument. This was achieved through a liquid lightguide connected to an angle-resolved platform and using a polarizer in the emission pathway. To measure the emission spectra, the exciton reservoir was excited at 2.403 eV and at a 15-degree angle normal to the surface. Emission from the cavities was recorded at angles ranging from 10 to 50 degrees in 5-degree intervals. Excitation and emission angles were kept orthogonal to each other to prevent specular reflection into the detector.

1.3 AFM Analysis

AFM was performed using AFM Cypher from Oxford instruments. The roughness analysis was carried out in tapping mode using Si tips with a tip radius of 10 nm for all measurements. AFM topographic images were recorded over scan areas of 20 μm x 20 μm (Figure S20).

2. Synthesis of the BODIPY Derivative

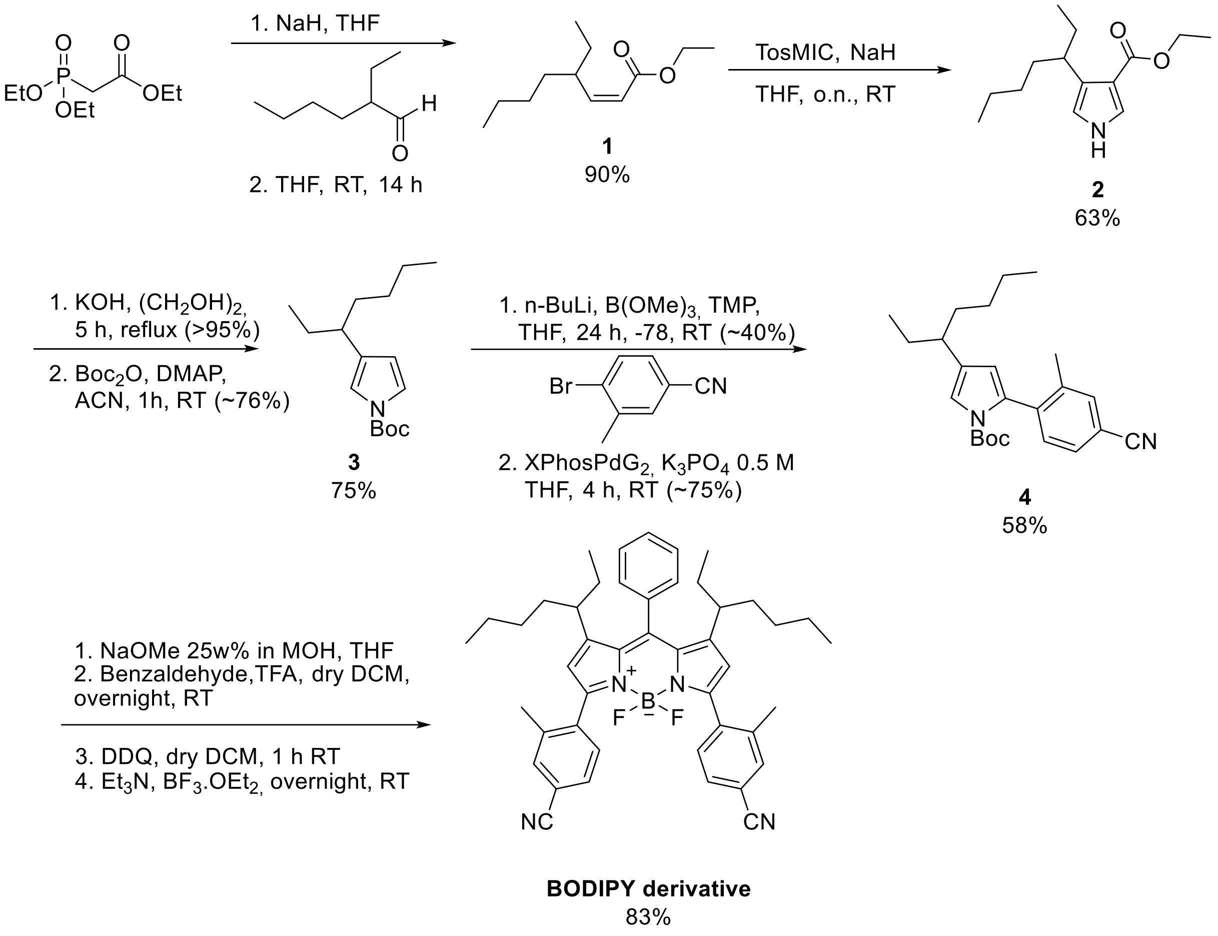

Scheme 1. Synthetic route towards the BODIPY derivative, following the previously reported route towards different alkylated BODIPY derivatives.[1]

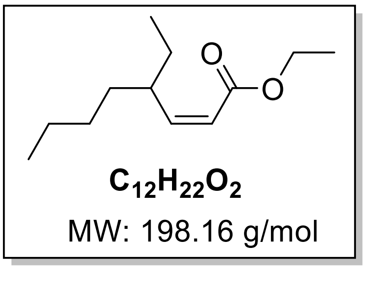

Compound **1**: This reaction was performed under N_2 atmosphere. Triethyl phosphono acetate (13.9 mL, 70.0 mmol, 1.40 eq) was added dropwise to a stirred solution of NaH (60% dispersion in mineral oil) (2.40 g, 60.0 mmol, 1.20 eq) in dry THF (30 mL) at room temperature. The resulting solution was stirred for 1 h before the aldehyde (8.05 mL, 51.5 mmol, 1.00 eq) in THF (30 mL) was added dropwise over 1 h at room temperature. The solution was stirred for 1 h, at ambient temperature. The reaction was quenched with NH_4Cl (100 mL) followed by extraction with diethyl ether (3 x 50 mL). The combined organic phases were washed with brine (2 x 50 mL) and dried over Na_2SO_4. Subsequently the solvent was removed under reduced pressure. Column chromatography on silica using 20 % diethyl ether in hexane as an eluent afforded the product as a clear oily liquid (9.2 g, 46.4 mmol, 90 %).

^{1}H NMR (600 MHz, $CDCl_3$) δ 6.73 (dd, *J* = 15.6, 9.3 Hz, 1H), 5.77 (dd, *J* = 15.6, 0.9 Hz, 1H), 4.19 (q, *J* = 7.1 Hz, 2H), 2.04 (dt, *J* = 9.2, 4.7 Hz, 1H), 1.52 – 1.40 (m, 2H), 1.38 – 1.32 (m, 2H), 1.30 (t, *J* = 7.1 Hz, 3H), 1.28 – 1.15 (m, 4H), 0.86 (dt, *J* = 15.0, 7.2 Hz, 6H). **^{13}C NMR** (151 MHz, $CDCl_3$) δ 166.8, 153.7, 121.1, 77.3, 77.0, 76.8, 60.2, 44.4, 33.8, 29.4, 27.2, 22.8, 14.3, 14.0, 11.7. **HRMS:** (ESI$^+$) m/z calcd. for (M) $C_{12}H_{22}O_2$: 198,16198; found: 198,1627.

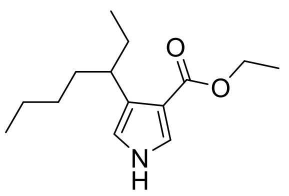

$C_{14}H_{23}NO_2$

MW: 237.34 g/mol

Compound **2**: This reaction was performed under N_2 atmosphere. A mixture of the **1** (8.90 g, 45.0 mmol, 1.00 eq) and TosMIC (9.17 g, 47.0 mmol, 1.05 eq) in dry diethyl ether/DMSO (90 mL/45 mL) was added dropwise to a stirred suspension of NaH (60 % dispersion in mineral oil) (3.14 g, 81 mmol, 1.7 eq) in diethyl ether (40 mL) at room temperature, for 1.5 h using a dripping funnel. The resulting suspension was stirred at room temperature for 5 h. Ice water was added to the reaction mixture. The aqueous phase was extracted with diethyl ether (3 x 50 mL). The combined organic phases were washed with brine, dried over Na_2SO_4 and the solvent was removed under reduced pressure. The crude product was purified using column chromatography on silica with 10 – 30 % ethyl acetate/hexane. The product was isolated as a light yellow solid (6.79 g, 28.6 mmol, 63 %).

^{1}H NMR (600 MHz, $CDCl_3$) δ 8.36 (s, 1H), 7.39 (dd, *J* = 3.1, 2.3 Hz, 1H), 6.51 (t, *J* = 2.3 Hz, 1H), 4.26 (q, *J* = 7.1 Hz, 2H), 3.19 (tt, *J* = 7.7, 5.8 Hz, 1H), 1.66 – 1.49 (m, 4H), 1.33 (t, *J* = 7.1 Hz, 3H), 1.31 – 1.20 (m, 4H), 0.83 (dt, *J* = 15.1, 7.2 Hz, 6H). **^{13}C NMR** (151 MHz, $CDCl_3$) δ 165.4, 130.4, 124.3, 115.8, 114.6, 77.2, 77.0, 76.8, 59.3, 36.9, 35.1, 29.6, 28.7, 23.0, 14.5, 14.1, 11.6. **HRMS:** (ESI^+) m/z calcd. for (M) $C_{14}H_{23}NO_2$: 237,17288; found: 237,1737.

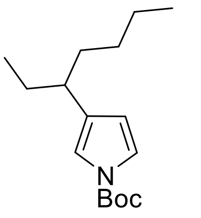

$C_{16}H_{27}NO_2$

MW: 265.20 g/mol

Compound **3**: Decarboxylation: **2** (5.94 g, 25 mmol, 1.00 eq) and KOH (7.00 g, 125 mmol, 5.00 eq) were put in a flask together with ethylene glycol (50 mL) and the reaction mixture was refluxed for 5 h. After cooling down to room temperature the reaction mixture was extracted with $CHCl_3$ (3 x 50 mL). The combined organic phases were washed with brine (2 x 75 mL), dried over Na_2SO_4 and the solvent was removed under reduced pressure. The crude products were used directly and without further purification for Boc-protection. The alkylated, unprotected pyrrole and Et_3N (7.0 mL, 50.0 mmol, 2.00 eq) was put in DCM (12.5 mL). Boc-anhydride (6.89 mL, 30.0 mmol, 1.20 eq), followed by DMAP (0.306 g, 2.50 mmol, 0.10 eq) was added to the solution. The reaction mixture was stirred at 30°C until full conversion (24 h). The solvent was removed under reduced pressure. The crude product was purified using column chromatography on SiO_2 (Hexane:EtOAc 1-10 %) and isolated as a yellow oil (4.98 g, 18.8 mmol, 75 %).

^{1}H NMR (600 MHz, $CDCl_3$) δ 7.15 (s, 1H), 6.95 (s, 1H), 6.05 (dd, *J* = 3.2, 1.7 Hz, 1H), 2.31 (td, *J* = 8.8, 4.6 Hz, 2H), 1.59 (s, 9H), 1.57 – 1.51 (m, 2H), 1.48 – 1.39 (m, 2H), 1.33 – 1.24 (m, 2H), 1.22 – 1.16 (m, 2H), 0.86 (t, *J* = 7.2 Hz, 3H), 0.81 (t, *J* = 7.3 Hz, 3H). **^{13}C NMR** (151 MHz, $CDCl_3$) δ 149.1, 131.8, 119.9, 116.6, 111.5, 83.1, 77.2, 77.0, 76.8, 39.4, 35.5, 29.7, 28.9, 28.0, 22.8, 14.1, 12.0.**HRMS:** (ESI^+) m/z calcd. for (M) $C_{16}H_{27}NO_2$: 265,20418; found: 265,2058.

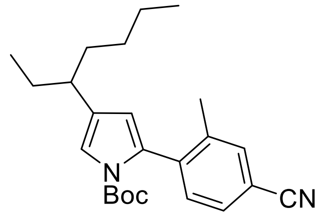

$C_{24}H_{32}N_2O_2$

MW: 380.53 g/mol

Compound **4:** This reaction was performed under N_2 atmosphere. A solution of 2,2,6,6-tetramethylpiperidin (2.75 g, 19.5 mmol, 1.20 eq) in dry THF (60 mL) was cooled to -78 °C. To the cooled solution, n-BuLi (2.5 M in hexanes) (9.00 mL, 22.5 mmol, 1.50 eq) was added dropwise over 30 min. The mixture was warmed to 0 °C. After stirring at this temperature for 30 min the mixture was cooled down to -78 °C again. **3** (3.98 g, 15.0 mmol, 1.00 eq) in dry THF (30 mL) was added dropwise over 30 min, and afterwards the reaction was left to stir at -78 °C for 2 h. Trimethylborate (7.79 g, 75.0 mmol, 5.00 eq) was added to the reaction mixture over 30 min. The resulting solution was left to warm up to room temperature where it was left to stir overnight. Afterwards, the reaction mixture was diluted with EtOAc. The organic phase was washed with H_2O (2 x 50 mL) and brine (50 mL) and subsequently dried over Na_2SO_4 and the solvent was removed

under reduced pressure. The crude product was crystallized in hexane. After crystallization the pyrrole boronic acid was immediately used in the following Suzuki coupling, without further analysis (1.88 g, 6.09 mmol, 40%).

The pyrrole boronic acid (1.88 g, 6.09 mmol, 1.00 eq), XPhosPdG2 (0.95 g, 0.12 mmol, 0.02 eq) and 4-Bromo-3-methylbenzonitrile (1.19 g, 6.09 mmol, 1.00 equivalent), were added to a vial, which was subsequently evacuated and refilled with N_2 (3 cycles). THF and 0.5 M K_3PO_4 were bubbled with N_2 for 1 h. THF (12 mL) was added to the solids. After the solids were dissolved K_3PO_4 (24 mL) was added. The reaction mixture was stirred for 6 h at room temperature. Conversion was followed via TLC (Hexane:EtOAc 10 %; stained with vanillin). H_2O and EtOAc were added to the reaction mixture and the phases were separated. The aqueous layer was extracted with EtOAc (2 x 20 mL). The combined organic phases were dried over Na_2SO_4 and the solvent was removed under reduced pressure. The crude product was purified using flash column chromatography (SiO_2 column, Hexane:EtOAc up to 10 %). The product was isolated as a light yellow-brownish solid (2.00 g, 5.26 mmol, 88%)

^{1}H NMR (600 MHz, $CDCl_3$) δ 7.47 (dd, *J* = 4.0, 2.5 Hz, 2H), 7.32 (d, *J* = 8.2 Hz, 1H), 7.10 (d, *J* = 1.9 Hz, 1H), 5.95 (d, *J* = 1.9 Hz, 1H), 2.35 (tt, *J* = 8.6, 5.3 Hz, 1H), 2.16 (s, 3H), 1.66 – 1.53 (m, 2H), 1.51 – 1.44 (m, 2H), 1.29 (s, 9H), 1.39 – 1.17 (m, 4H), 0.90 – 0.82 (m, 6H). **^{13}C NMR** (151 MHz, $CDCl_3$) δ 149.0, 140.5, 139.4, 132.6, 131.3, 130.7, 130.5, 129.0, 119.1, 118.7, 114.3, 111.2, 83.4, 77.3, 77.0, 76.8, 39.3, 35.3, 31.6, 29.7, 28.8, 27.6, 22.8, 22.7, 19.7, 14.1, 12.0. **HRMS:** (ESI$^+$) m/z calcd. for (M) $C_{24}H_{32}N_2O_2$: 380,24638; found: 380,2487.

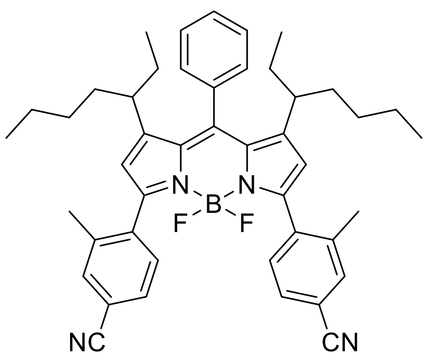

$C_{45}H_{49}BF_2N_4$
MW: 694.72 g/mol

BODIPY derivative: 4 (1.90 g, 5.00 mmol, 2.00 eq) was added to the reaction vessel, which was subsequently evacuated and refilled with N_2 (3 cycles). The starting material was dissolved in dry THF (5 mL·mmol^{-1}). NaOMe (25 w % in MeOH, 2 mL) was added to the reaction mixture and the reaction was stirred until full conversion of the starting material (1.5 h). H_2O (30 mL) was added to the reaction mixture and the phases were separated. The aqueous phase was extracted with diethyl ether (2 × 20 mL) and the combined organic phases were washed with brine (25 mL). The organic phase was dried overNa2SO4 and the solvent was subsequently removed under reduced pressure. Deprotected **4** was obtained as a light-yellow oil and was directly used without further purification or analysis. Deprotected **4** and benzaldehyde (265 mg, 2.5 mmol, 1.00 eq) were dissolved in DCM_{dry} (250 mL). A catalytic amount of TFA (0.2 mL) was added to the solution and the solution was stirred overnight, until TLC showed full conversion of benzaldehyde (TLC: SiO_2 hexane/EtOAc 10%). After full conversion was determined, DDQ (1.15 g, 5.10 mmol, 2.05 eq) in DCM (60 mL) was added to the reaction mixture. The reaction mixture, which changed color from dark pink to dark purple, was stirred at room temperature for 1 h. Et_3N (16 mL) was added to the reaction mixture, accompanied by a color change to dark red, and stirred for 30 min at room temperature. Afterward $BF_3{\cdot}OEt_2$ (16 mL) was added to the reaction mixture and the now dark purple solution was stirred overnight. H_2O was added to the reaction mixture, and the two-phase mixture was stirred for 1 h. There after the phases were separated, and the organic phase was washed with H_2O (6 × 150 mL), afterwards dried over Na_2SO_4, and then the solvent was removed under reduced pressure. The crude product was first purified by column chromatography (SiO_2 column, eluent: hexane/EtOAc 10-20 %), where the red band was collected. The collected red solid was purified further using column chromatography (SiO_2 column, eluent: hexane/Diethyl ether 5-20 %), where only the middle part

of the broad red band was collected. The product was obtained as a red crystalline solid (1.44 g, 2.07 mmol, 83 %).

^{1}H NMR (600 MHz, $CDCl_3$) δ 7.66 – 7.41 (m, 11H), 6.16 (s, 2H), 2.25 (s, 6H), 1.33 – 1.07 (m, 14H), 0.99 (tdd, *J* = 11.1, 6.3, 2.4 Hz, 2H), 0.88 – 0.84 (m, 2H), 0.81 (td, *J* = 7.3, 1.8 Hz, 6H), 0.60 (td, *J* = 7.2, 1.7 Hz, 6H). **^{13}C NMR** (151 MHz, $CDCl_3$) δ 155.3, 154.9, 145.7, 138.3, 137.7, 134.1, 133.1, 132.6, 130.7, 129.5, 129.3, 129.2, 129.1, 128.7, 128.4, 128.4, 128.3, 119.0, 118.8, 112.6, 77.2, 77.0, 76.8, 38.2, 35.8, 29.8, 29.6, 22.9, 20.1, 14.0, 11.9. **HRMS:** (ESI^+) m/z calcd. for $(M+Na)^+$ $C_{45}H_{49}BF_2N_4Na$: 717.39161; found: 717.3967.

3. Supplementary Analysis

3.1 Excitonic Energy and Homogenous Broadening Calculations

The solid-state absorption spectrum of the BODIPY derivative exhibit two peaks, at 2.385 eV and at 2.524 eV. These peaks are likely associated with vibronic energy levels of the same electronic transition. To determine the average electronic transition energy, the absorption spectrum was deconvoluted using two Voigt functions centered at 2.385 eV and 2.524 eV (Figure S8); having integrated areas of 0.1392 and 0.0665, respectively. In this fit the Lorentzian FWHM was 34 meV and 42 meV for the 2.385 eV and 2.524 eV peaks, respectively. The FWHM of the Lorentzian part of the function was considered as the homogenous broadening of the molecule.[2, 3] Subsequently, the average excitonic energy was calculated using Equation S1 to 2.43 eV.

$$E_X = \frac{\sum_{i=1}^{2} E_{\mathrm{V}i} A_i}{\sum_{i=1}^{2} A_i} \qquad \text{(S1)}$$

Where E_{x} is the excitonic energy, A_i is the area of the ith peak and $E_{\mathrm{V}i}$ is the energy of the ith peak in the absorption spectrum.

3.2 Emission Modelling

To simulate the emission from microcavities, we implement the transfer matrix method (TMM). In the first step of the modelling, we needed to determine the precise thicknesses and dielectric permittivities of all the layers in the cavities. This was done by fitting the experimental reflectivity of all three cavities using TMM. To perform the fitting procedure, we varied 12 unknown parameters: six correspond to the dielectric permittivity of Ag, which was modeled according to the Drude-Lorentz model: $\epsilon(\omega) = \epsilon_\infty - \frac{f_0^2}{\omega(\omega+i\gamma)} + \frac{f_1^2}{\omega_1^2-\omega^2-i\omega\Gamma}$ (the parameters were ϵ_∞, f_0, γ, f_1, ω_1, Γ). The other free parameters of the model were the thicknesses of the first mirror and the active layer. These parameters are specific for each cavity, so their total number adds up to six. The thickness of the back mirror was set to 100 nm. The dielectric permittivity of the active layer was obtained through ellipsometry measurements and is shown in Figure S12a. In Figure S12b, we render the dielectric permittivity of the Ag obtained through fitting the experimental cavity reflectivity, compared with the standard Drude model for Ag.[4] The reflectivity obtained by fitting using TMM are presented in Figure S13-15.

The obtained sample properties were then used to simulate the emission from the microcavities. To demonstrate the approach, let us consider a thin sublayer of the molecular layer consisting of randomly oriented dipoles (Figure S16). For simplicity, we will consider only three possible directions of the dipole moment (aligned with the x, y, and z axes); all other orientations are taken into account implicitly by summing over the signal from the three dipoles aligned with these basis directions.

A dipole within a sublayer at the coordinate z absorbs the excitation light with probability $\propto A(z) \cdot L_i$ (i=x,y,z). Where $A(z)$ essentially represents the molecular absorption by the layer and can be retrieved from TMM calculations, and L_i is the geometrical factor determining the relative efficiency of excitation for differently oriented dipoles. For the subsequent emission, however, the excitation geometrical factor does not play any role, because molecules with different polarization exchange energy relatively fast. So, we assume that molecules with different polarizations emit with equal probability what means $L = \frac{1}{3}(1,1,1)$.

Excited dipoles then emit their characteristic radiation pattern inside the cavity. The propagation of the dipole radiation through the layered structure can be described using the source terms method.[5] The key step is to divide the microcavity into two parts (top and bottom sides). Then, having obtained the solutions for the top and bottom sides of the structure separately using TMM,

we stitch them together. The final formula for the electric field leaving the cavity from the top side is as follows:

$$E_t = \frac{t_t(r_b A_b - A_t)}{1 - r_t r_b}, \quad \text{(S2)}$$

where r_i and t_i are the amplitude reflection and transmission coefficients of the top part (i=t) and of the bottom part (i=b). A_t, A_b represent the source terms, which depend on the polarization of the dipoles (See Table S3). The final expression essentially represents the sum of contributions from the dipoles emitting in the downward direction reflected from the bottom part of the nanostructure and the contribution of the upward emitting dipoles. Moreover, it linearly depends on the transmission of the top nanostructure since we detect the emission from the top side. The denominator essentially represents the standard cavity phase factor, giving the enhancement and inhibition effects.

Importantly, the values for the source terms are slightly different from the ones found in Ref. [5]. In particular:

1) the numerical prefactor for all the coefficients is $\sqrt{\frac{3}{8\pi}}$, since in this work we assume the dipoles direction to be random (so azimuthal and polar angles are random), while the coefficients in Ref. [5] correspond to the case when only the azimuth of the dipole is random.

2) The relative signs of A_t, A_b are different. The signs of the source coefficients in this work were chosen so the model correctly reproduces analytical results in case of 1 interface.[6, 7]

The last ingredient of our method is detection. According to the scheme in Figure S16, the light emitted by a dipole will be detected with the relative probability D_i^{pol} ($D^{TE} = (0,1,0), D^{TM} = (\cos^2\theta, 0, \sin^2\theta)$).

The described procedure can be applied for every detection angle and frequency separately. The final transmission function can be found as an integration over the sublayer position and summation over the orientation of the dipoles. To retrieve the emission pattern, we must multiply the transmission obtained in the previous step by the emission spectrum of the bare molecules. The final expression can be found in the main text (Eq. 4) The results obtained by the described procedure are presented in Figures S17-19.

The calculated emission matches well with the experimental polaritonic emission in terms of energy- and intensity of the TE and TM dispersion if no intermolecular energy transfer is present, and matches even better if intermolecular energy transfer is present, i.e., if intramolecular energy transfer is assumed to be fast enough compared to emission to remove any pump-induced inhomogeneity in the orientation of the excited molecules, thus resulting in the transition dipoles of the emitting molecules to be oriented randomly in the cavity.

4. Supplementary Figures

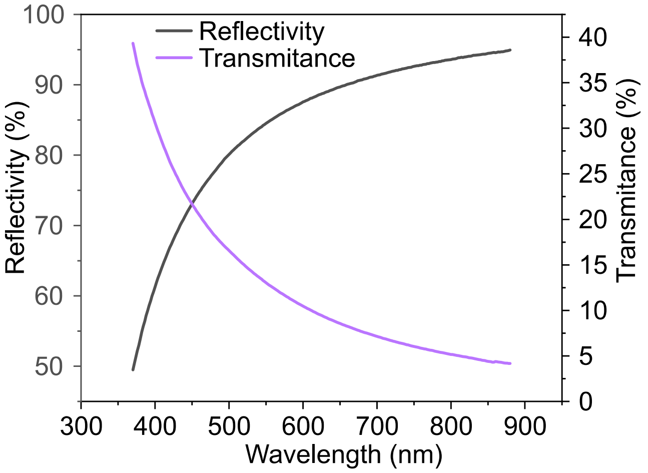

Figure S1. Reflectivity (black), and transmittance (purple) spectra of a 30 nm Ag mirror measured at 10 degrees.

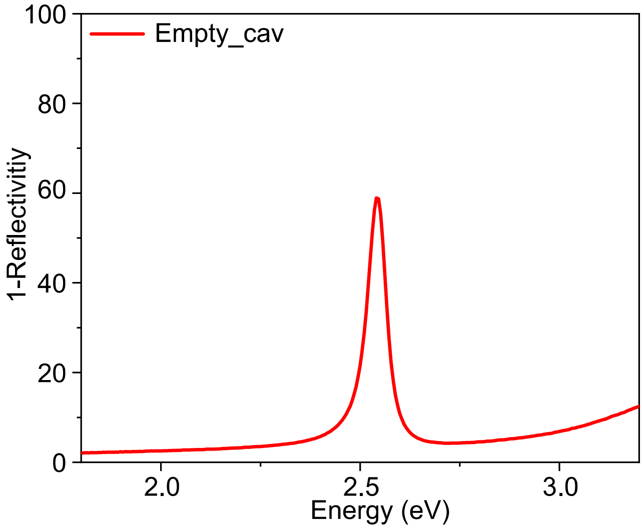

Figure S2. 1-Reflectivity spectrum of an empty cavity (PVA filled) having the same structure as the cavities filled with the BODIPY derivative. The FWHM of the cavity mode is 42 meV, corresponding to a quality factor of 60.

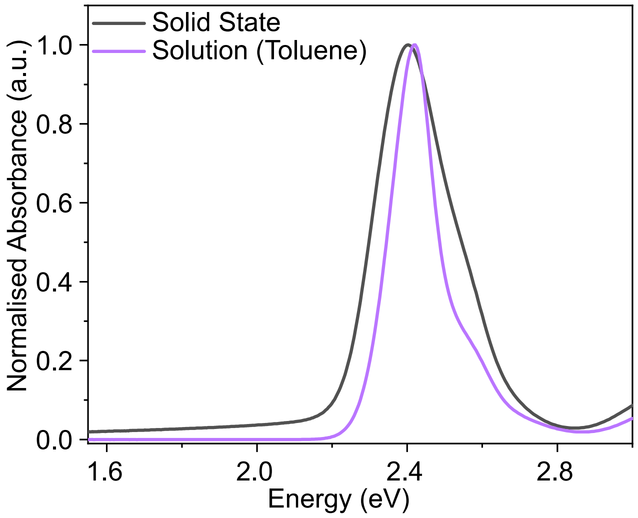

Figure S3. Absorbance of the BODIPY derivative in the solid state (black), and in toluene solution (purple).

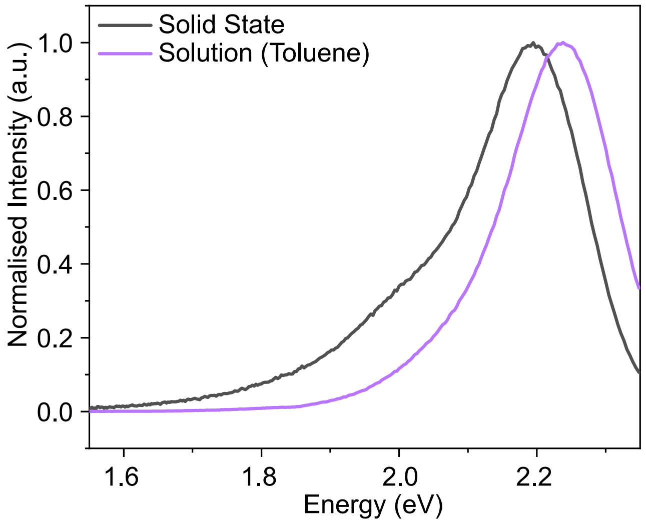

Figure S4. Emission of the BODIPY derivative in the solid state (black), and in toluene solution (purple).

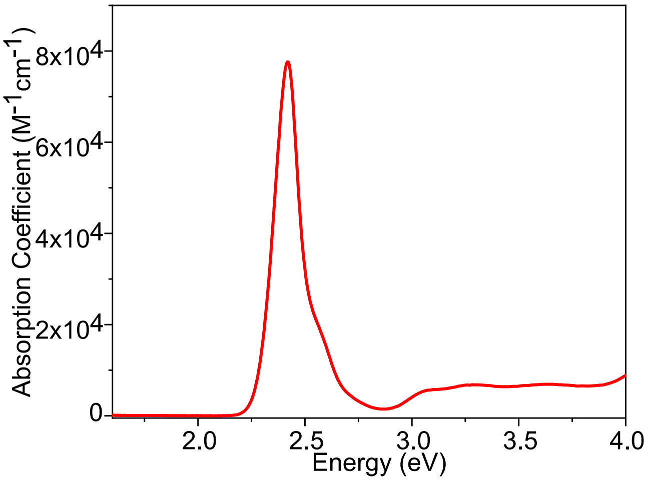

Figure S5. Absorption coefficient of the BODIPY derivative in toluene solution.

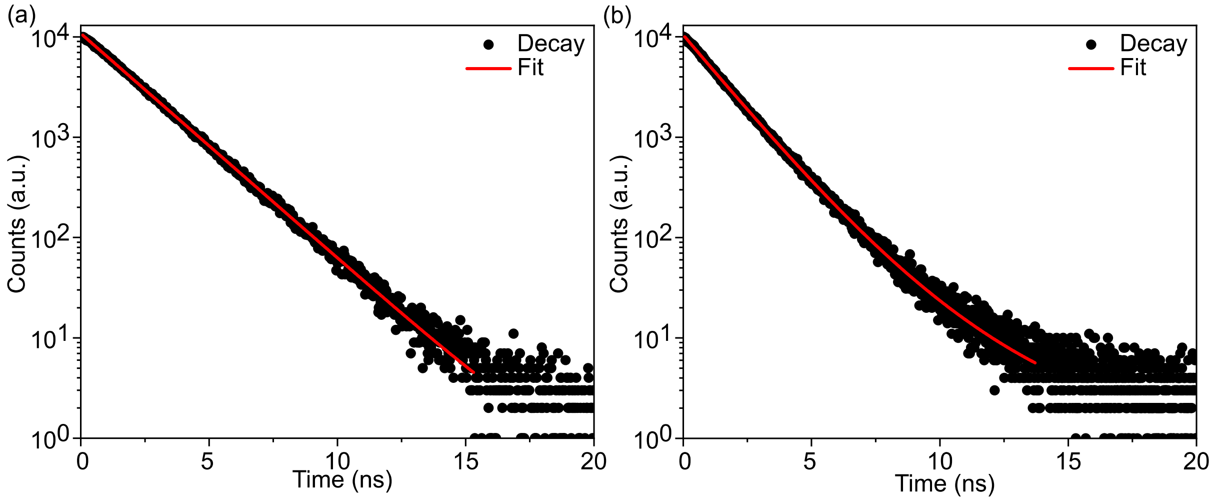

Figure S6. Excited state lifetime decay of the BODIPY derivative when exciting at 510 nm and recording the emission at 550 nm, measured in (a) toluene solution, (b) solid state.

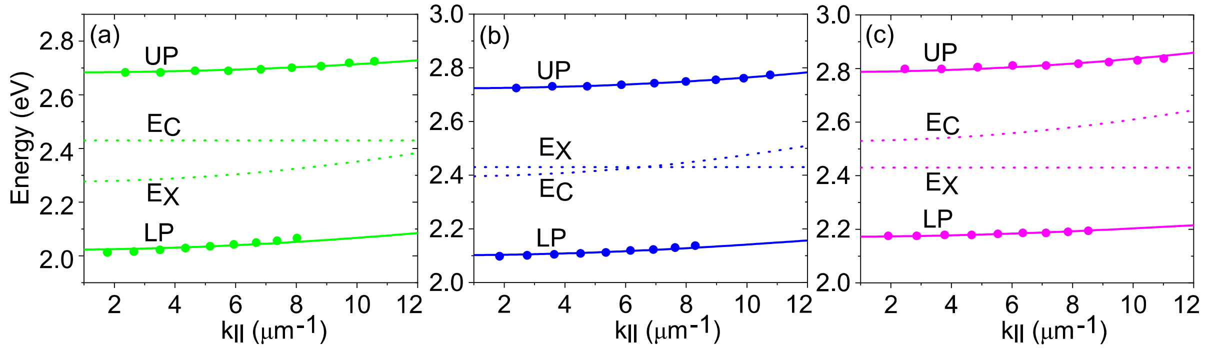

Figure S7. Energy of the upper and lower polaritons (taken as the reflectivity minima) as a function of in-plane momentum for the transverse magnetic (TM) polarization. Here, the molecular transition were coupled to cavities having a λ/2 mode with $E_c(0)$ at (a) 2.277 eV, (b) 2.395 eV, and (c) 2.529 eV. The solid circles correspond to the experimental polaritonic energies, the solid lines represent the fit of the polaritonic dispersion using the coupled harmonic oscillator model, and the dotted lines corresponds to the energies of the molecular transition and cavities.

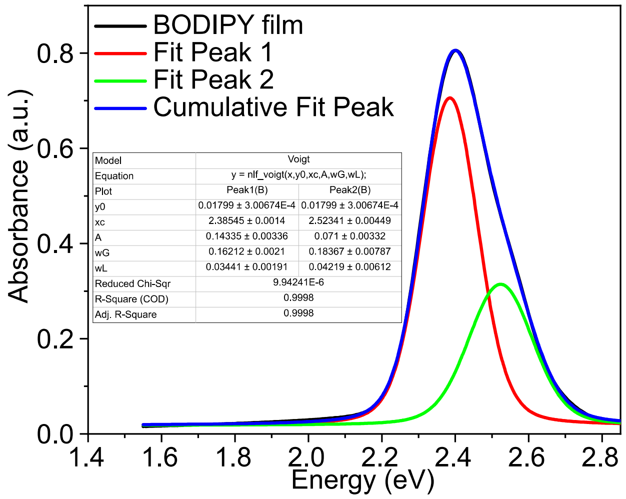

Figure S8. Deconvolution of the molecular absorption spectrum using two Voigt functions, having peak energies at 2.385 eV (red), and 2.524 eV (green).

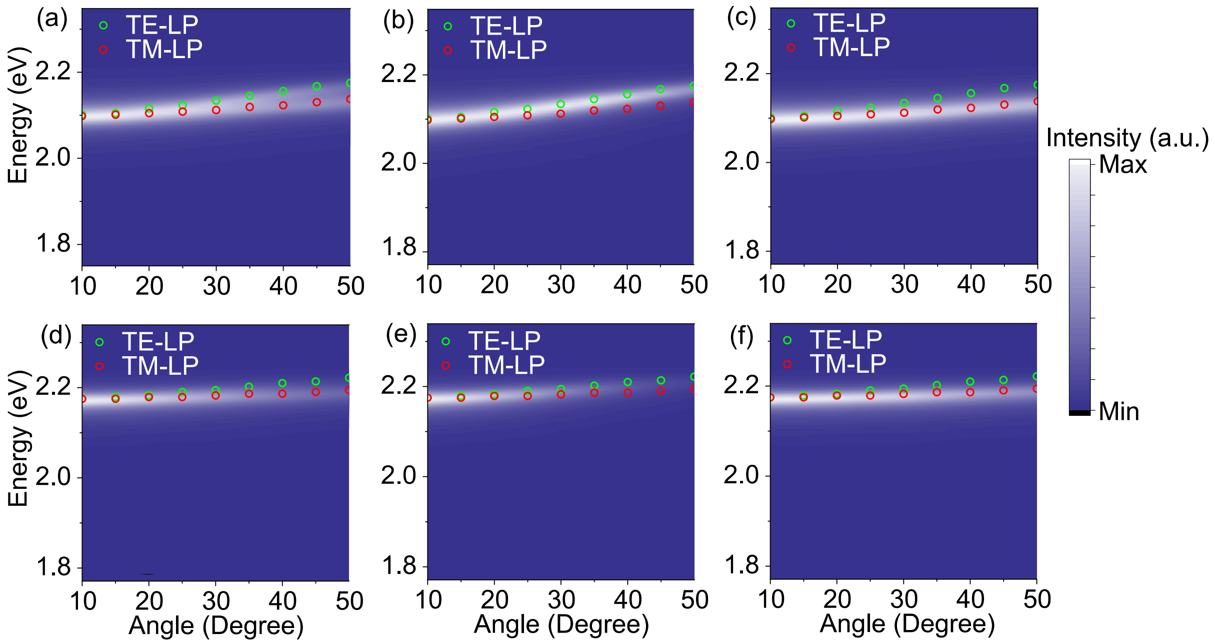

Figure S9. Angle-dependent emission contour plots for the cavity having a $E_c(0)$ = 2.395 eV. The excitation energy was 2.403 eV (thus non-resonantly) and was performed at an angle of 15°. To avoid reflection into the detector, the emission was captured orthogonal to the plane of excitation. The green and red circles correspond to the reflectivity minima of the lower polariton in the transverse electric (TE) and transverse magnetic (TM) polarizations, respectively. The emission spectra were recorded (a) isotropically, (b) in TE polarization, and (c) in TM polarization. In the plots, white indicate low emission and black indicated maximum emission intensity. Additionally, emission spectra for another cavity, characterized by a λ/2 mode order and a cavity energy ($E_c(0)$ = 2.529 eV), were recorded (d) isotropically, (e) in TE polarization, and (f) in TM polarization.

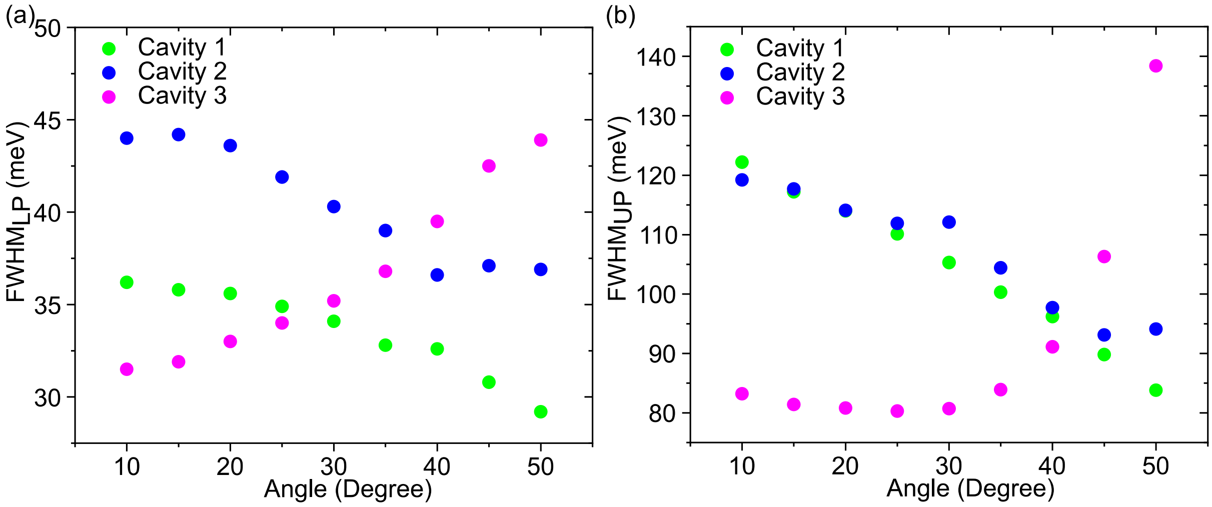

Figure S10. Full width at half maxima of absorptivity peak of the lower polariton (a), and upper polariton (b) measured in transverse electric (TE) polarized light. Green, blue, and pink circles correspond to cavities with energies (E_C (0)) of 2.277 eV, 2.395 eV, and 2.595 eV. Homogenous broadening of the molecular transition was 34- 42 meV. Full width at half maximum of the cavity mode was 42 meV. The theoretically minimum linewidth for the polariton is the average of the molecular transition and cavity mode, which equals to 38-42 meV.

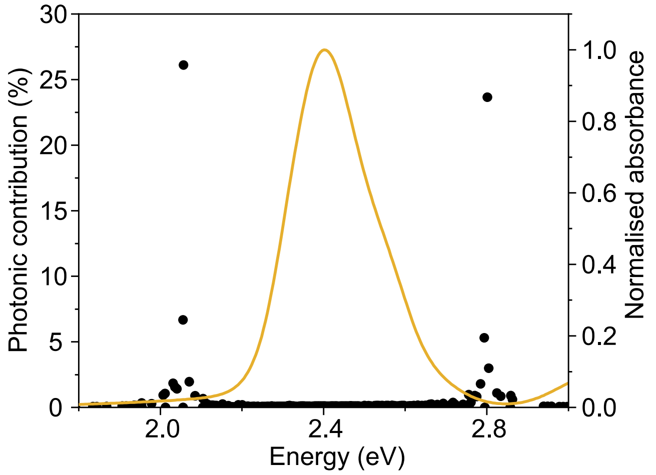

Figure S11. The photonic contribution of polaritonic states in a Tavis-Cummings model calculated using 1000 molecular states and 1 photonic mode for the cavity having a cavity energy of 2.42 eV and a linewidth of 100 meV, with the molecules randomly distributed in energy according to their absorption spectrum (shown in yellow), and a collective Rabi splitting of 0.7 eV. The figure shows that the only states with significant cavity contribution are centered around the polariton frequencies, and the "dark states" at the bare-molecule energies have negligible cavity contribution.

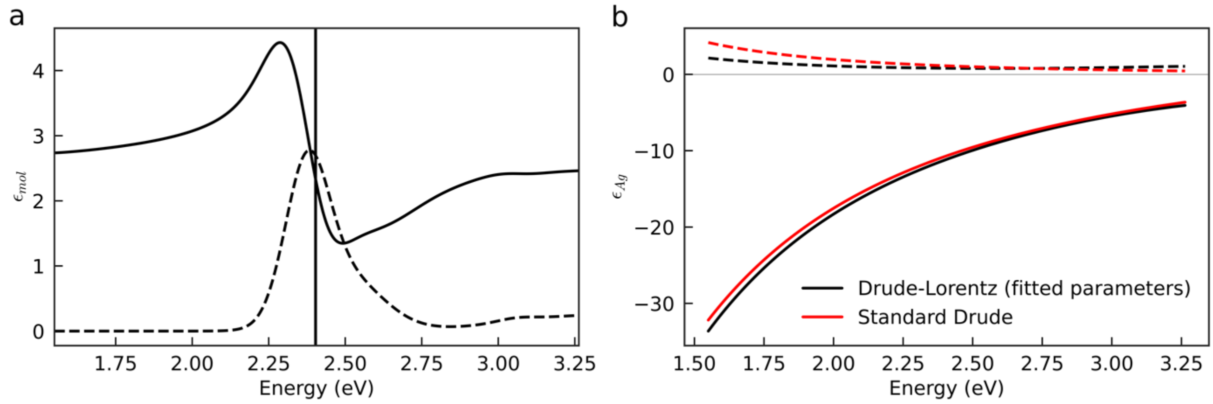

Figure. S12. The dielectric permittivity of (a) the BODIPY molecular layer, (b) an Ag film. The dashed line represents the imaginary part of the dielectric permittivity, and the solid line depicts the frequency dependence of the real part.

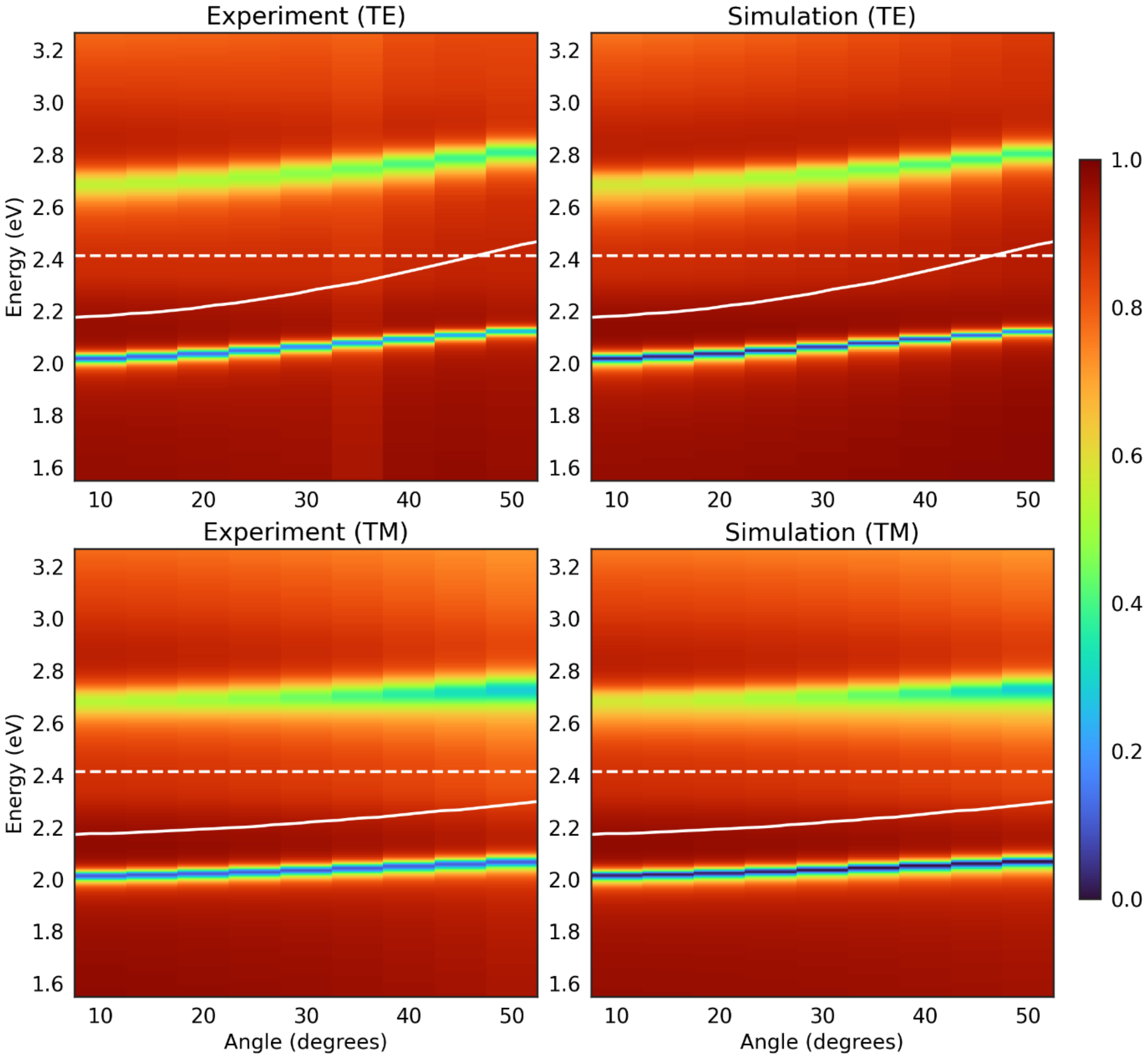

Figure S13. Reflectivity of the cavity with a cavity energy of $E_c(0)$ = 2.277 eV. The white solid line shows the position of the bare cavity mode, and the dashed line corresponds to the maxima of the BODIPY absorption.

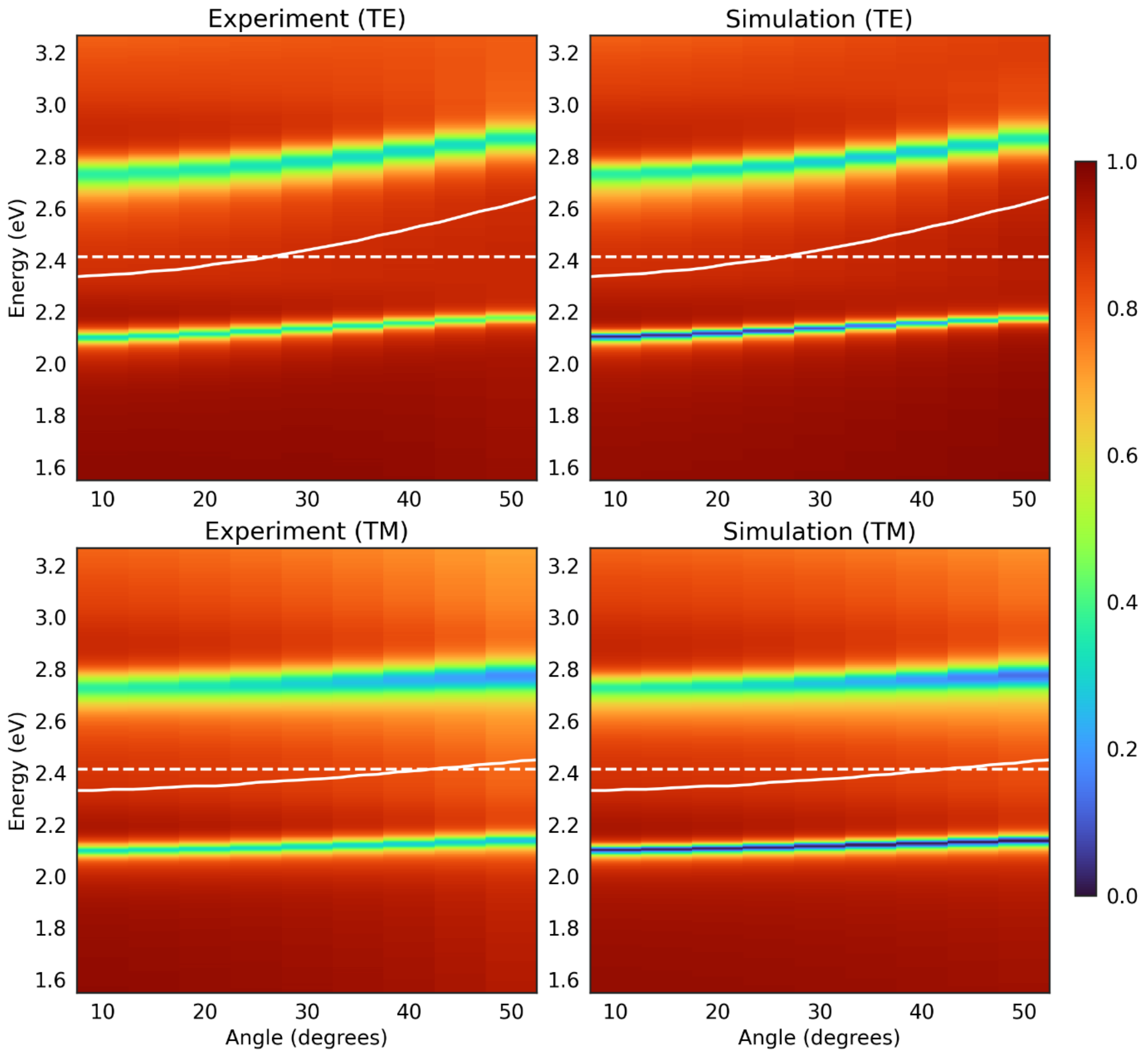

Figure S14. Reflectivity of the cavity with a cavity energy of $E_c(0)$ = 2.395 eV. The white solid line shows the position of the bare cavity mode, and the dashed line corresponds to the maxima of the BODIPY absorption.

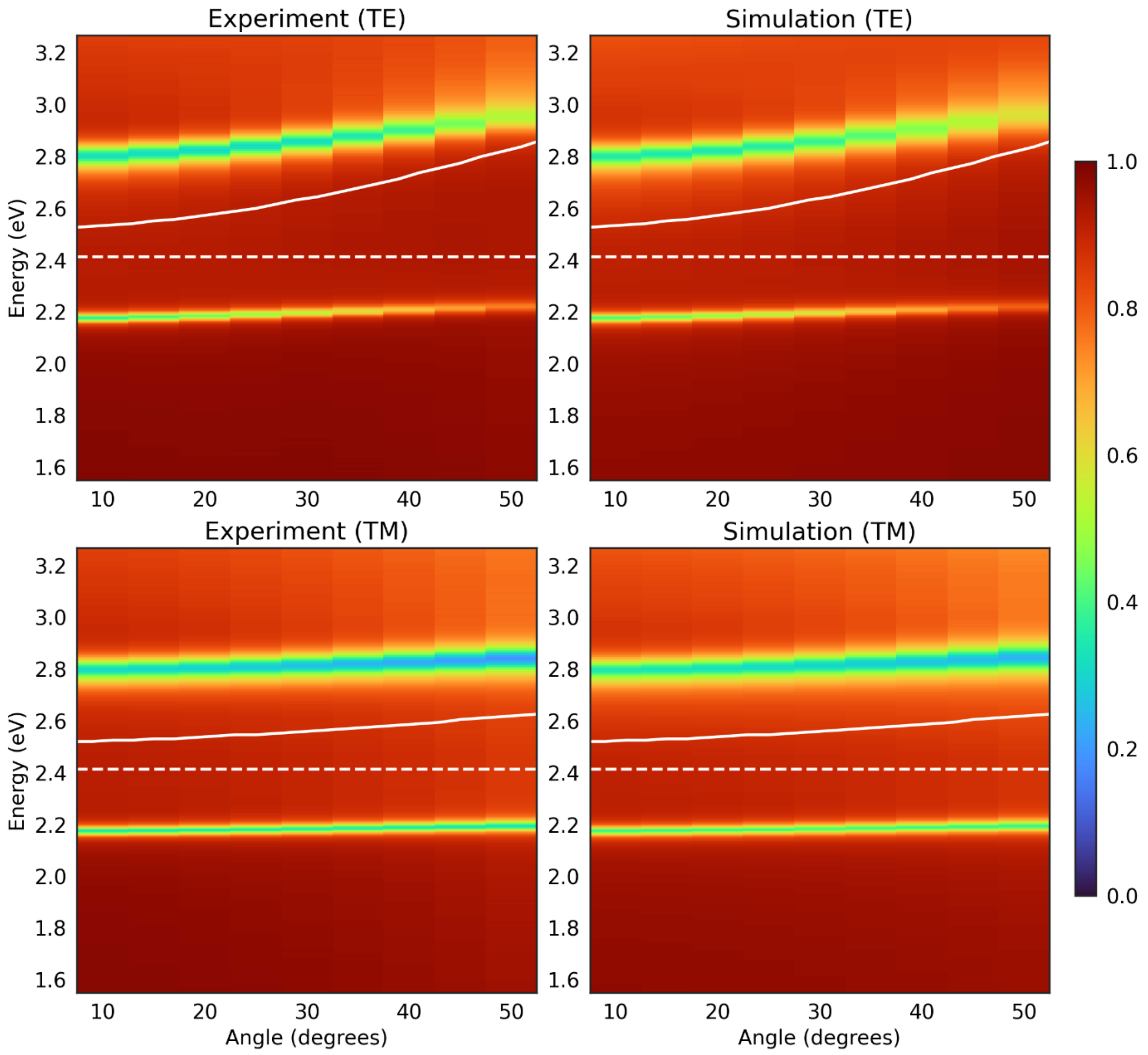

Figure S15. Reflectivity of the cavity with a cavity energy of $E_c(0)$ = 2.529 eV. The white solid line shows the position of the bare cavity mode, and the dashed line corresponds to the maxima of the BODIPY absorption.

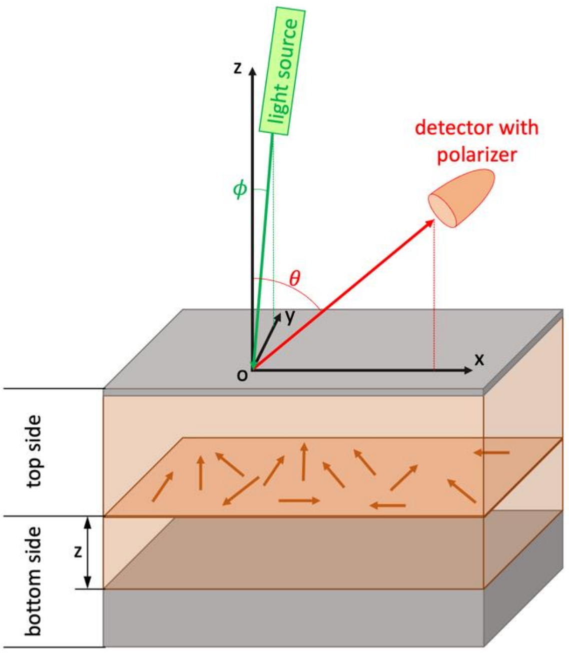

Figure S16. Schematic sketch of the experimental setup for the angle dependent emission measurements.

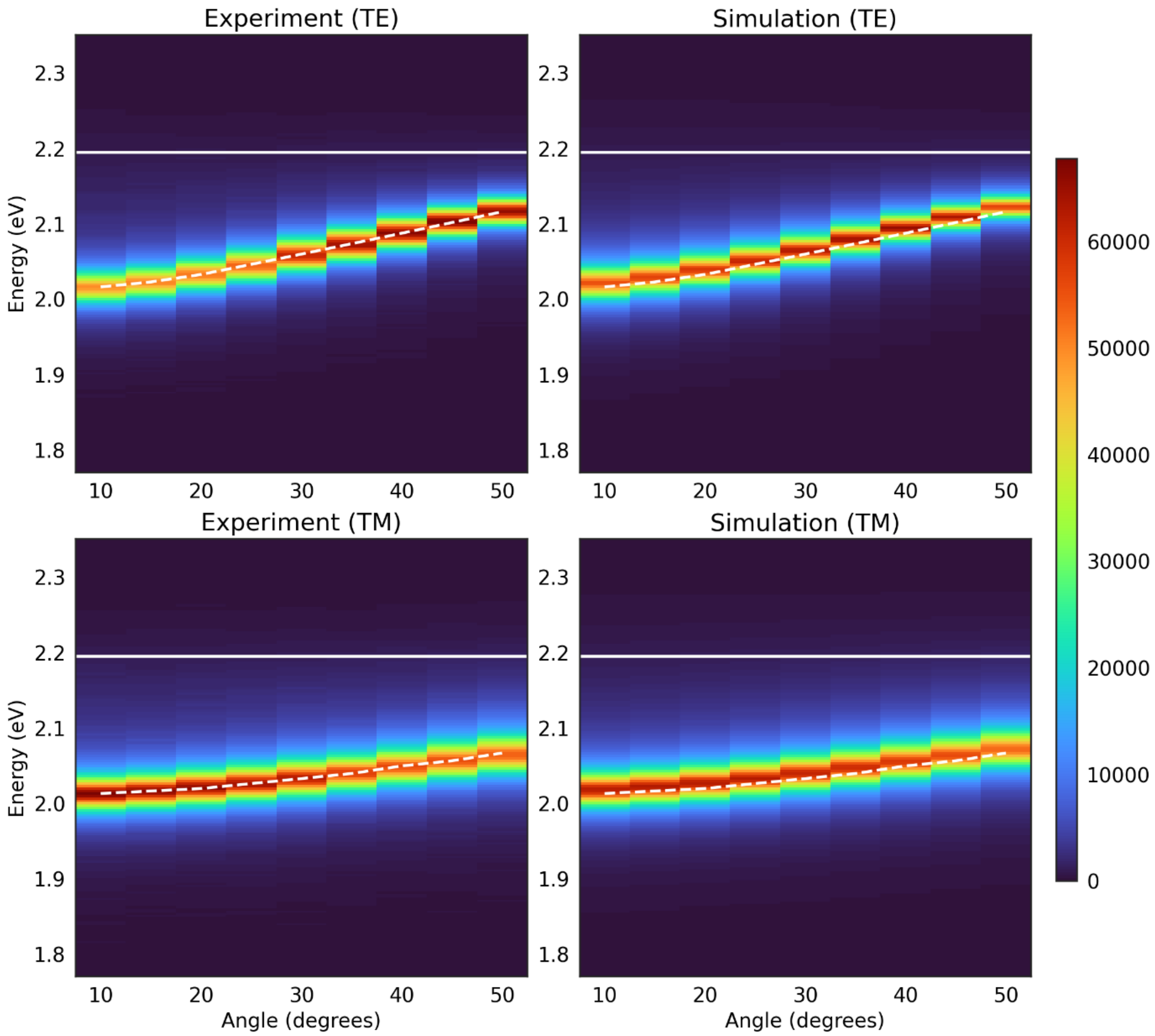

Figure S17. Experimental and simulated emission from the cavity with the mode energy $E_c(0)$ = 2.277 eV. The white solid lines show the maxima of the BODIPY derivative emission band, and the dashed lines correspond to the experimental position of LP.

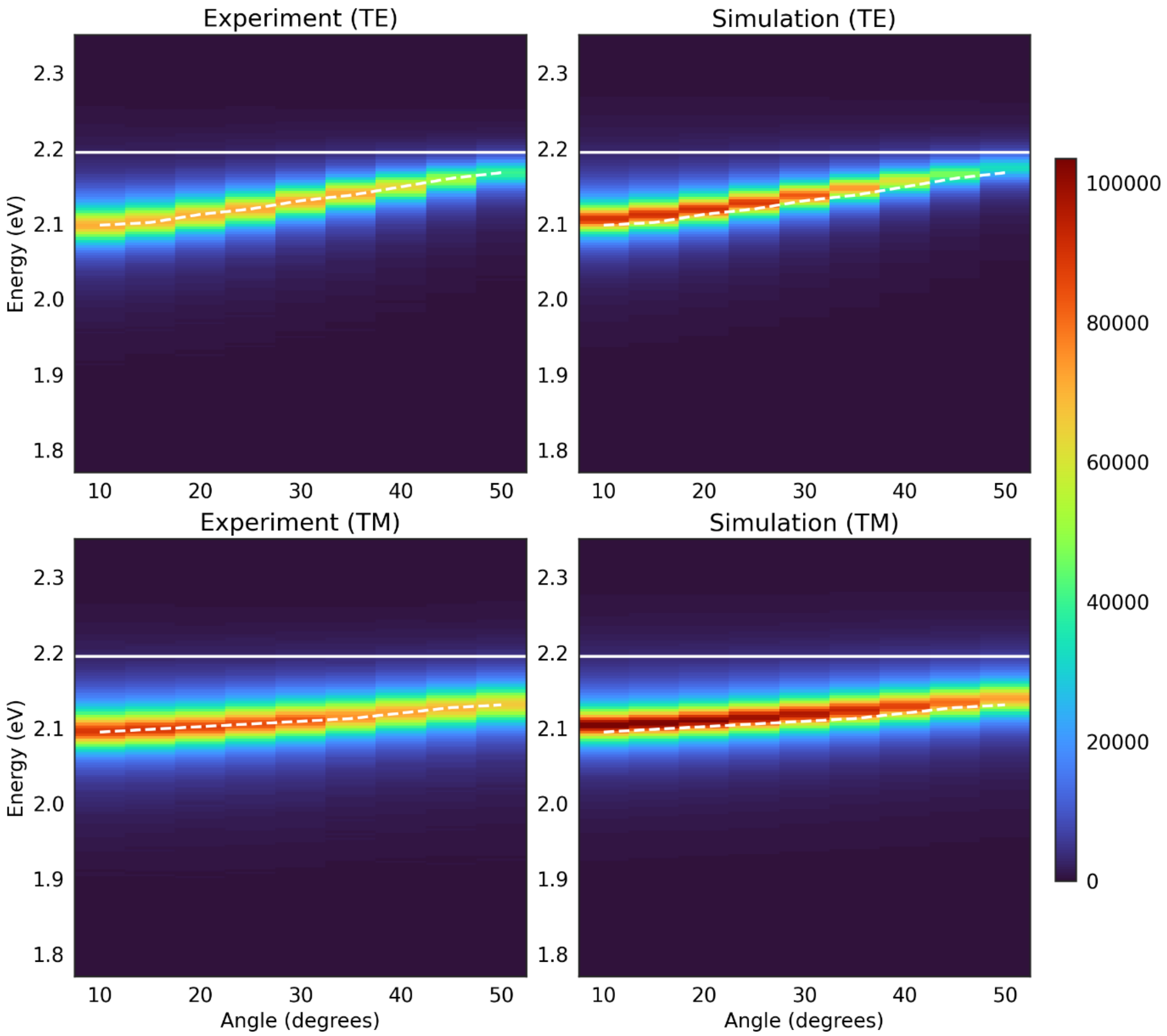

Figure S18. Experimental and simulated emission from the cavity with the mode energy $E_c(0)$ = 2.395 eV. The white solid line shows the peak of the BODIPY derivative emission band, and the dashed line corresponds to the experimental position of LP.

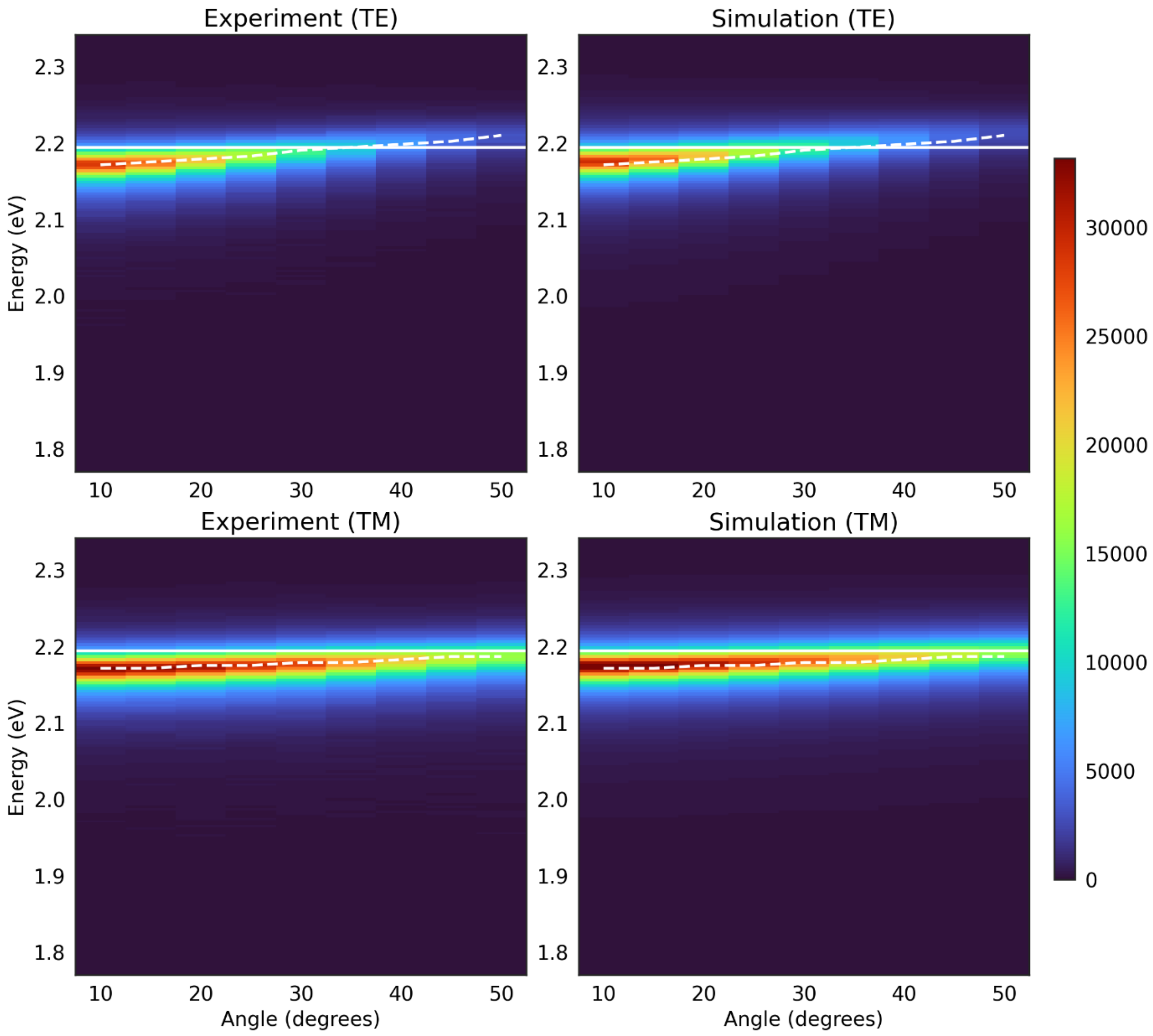

Figure S19. Experimental and simulated emission from the cavity with the mode energy $E_c(0)$ = 2.529 eV. The white solid line shows the peak of the BODIPY derivative emission band, and the dashed line corresponds to the experimental position of LP.

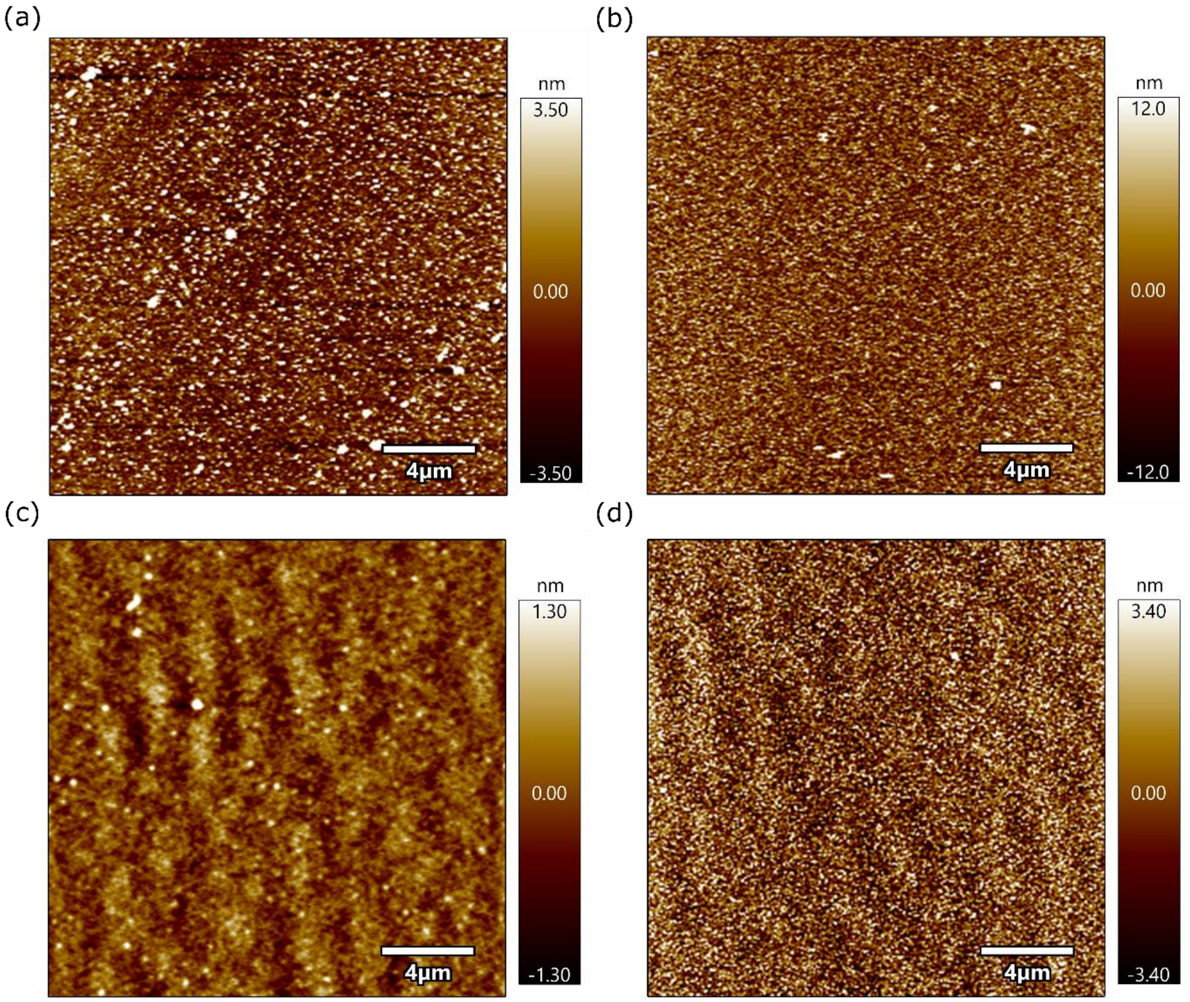

Figure S20. AFM analysis of all the surface layers of a cavity. (a) Glass substrate, (b) 100 nm Ag layer on the glass substrate, (c) the BODIPY derivatives molecular layer on top of the 100 nm Ag layer, and (d) the 30 nm Ag layer on top of the molecular layer. The surface roughness RMS value of each cavity layer was calculated to 1.5 nm (glass substrate), 4.5 nm (100 nm Ag layer), 0.3 nm (BODIPY derivative molecular layer), and 1.7 nm (30 nm Ag layer).

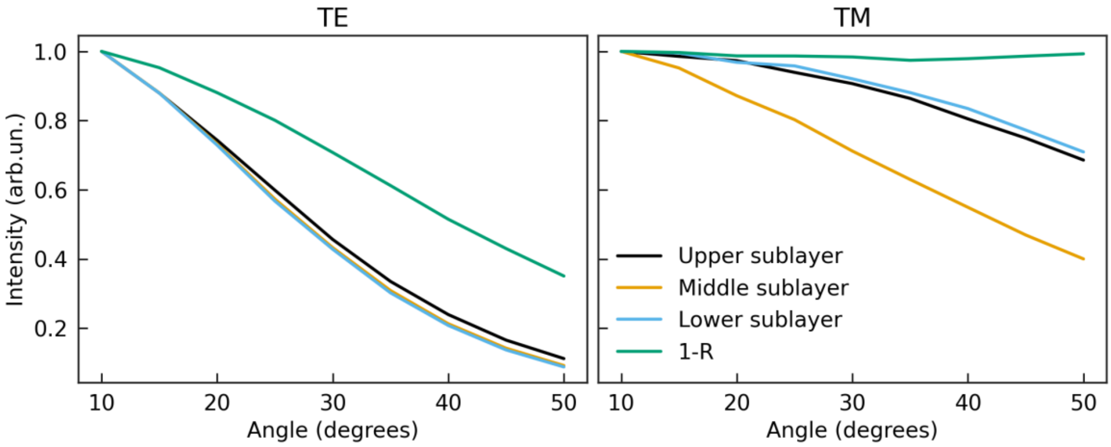

Figure S21. Signal transmission at the LP frequency from different points in the molecular layer of the cavity ($E_c(0)$= 2.529 eV).

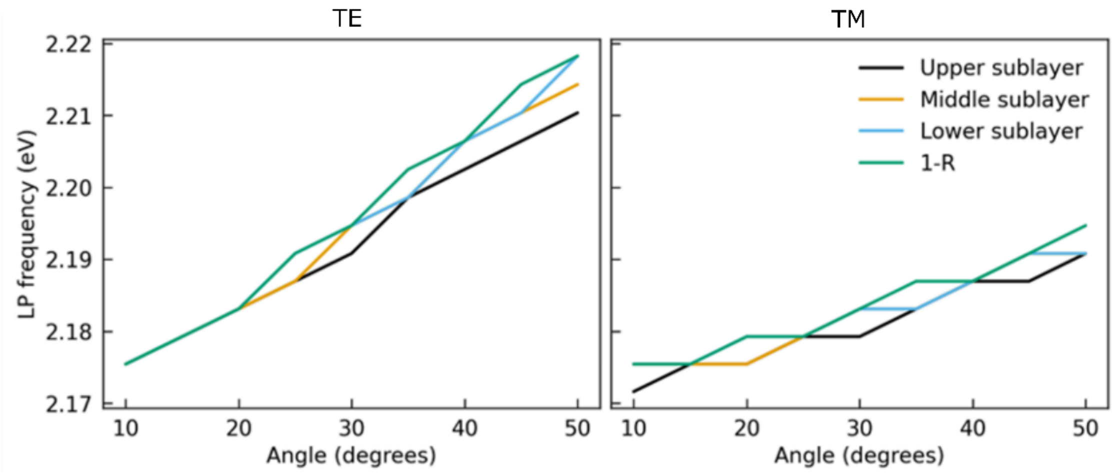

Figure S22. Lower polariton dispersion from different points in the molecular layer of the cavity ($E_c(0)$= 2.529 eV).

5. ^{1}H and ^{13}C NMR

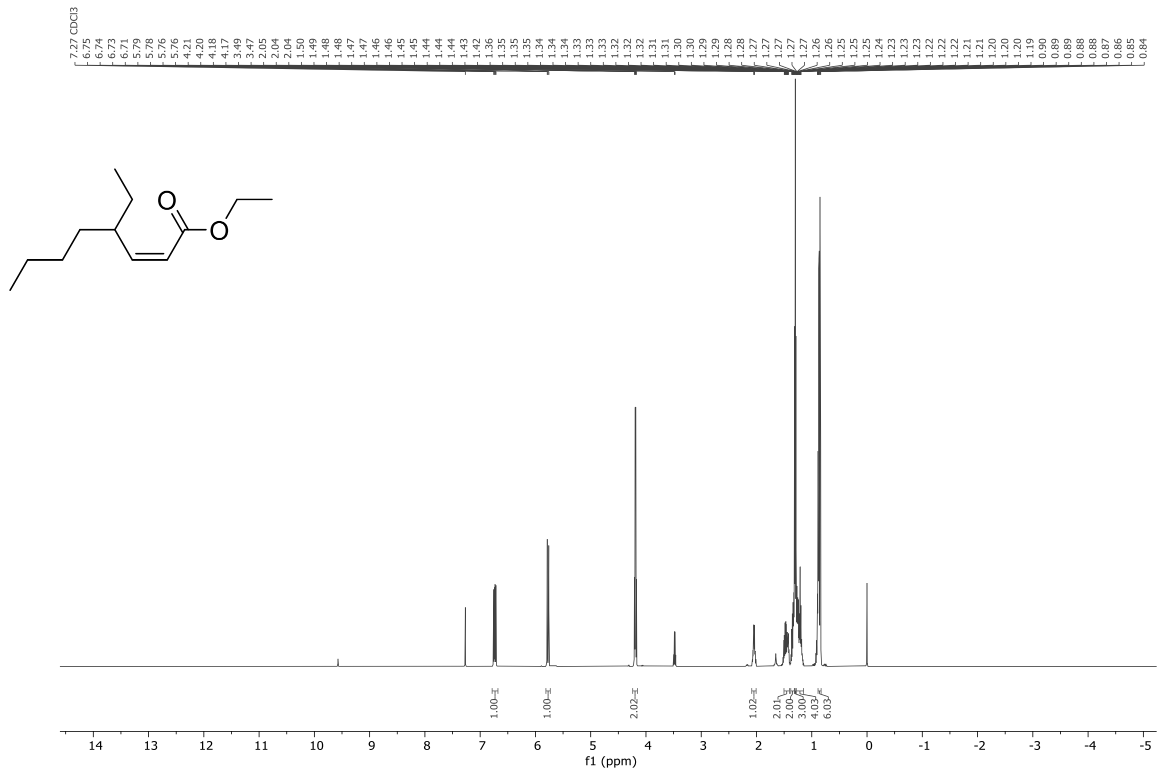

Figure S23. ^{1}H-NMR (600 MHz, $CDCl_3$) spectrum of **1.**

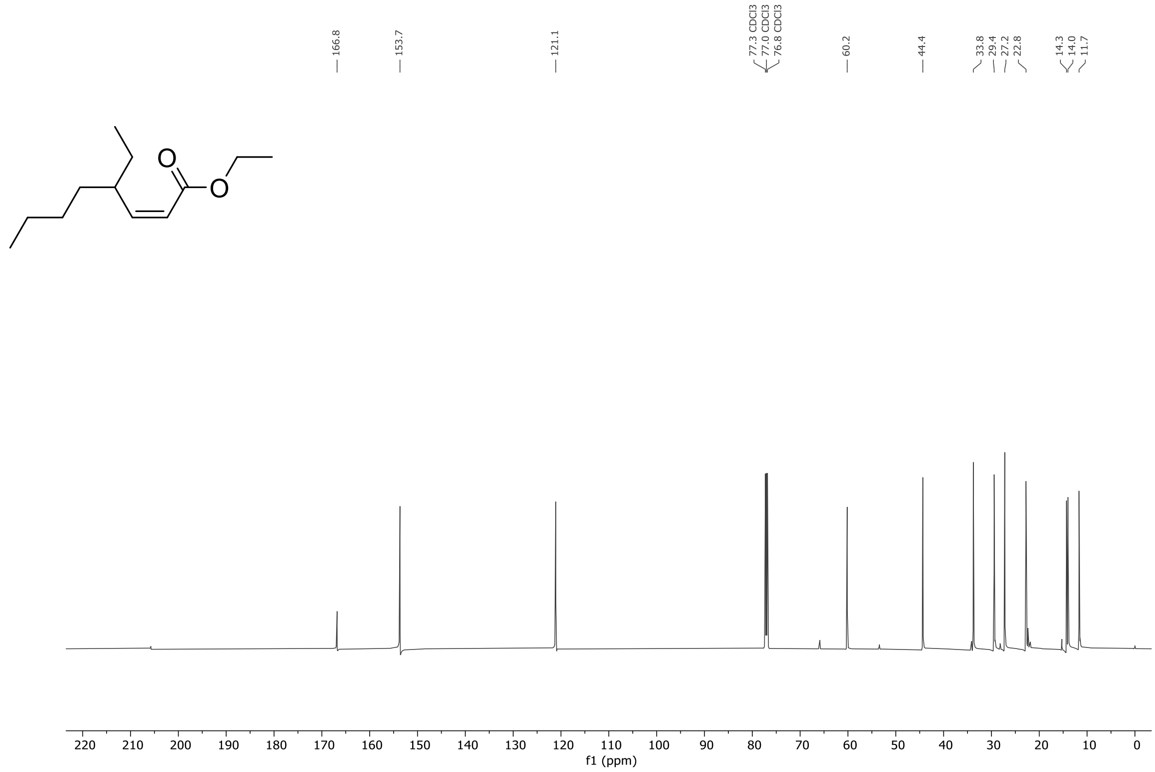

Figure S24. ^{13}C-NMR (600 MHz, $CDCl_3$) spectrum of **1.**

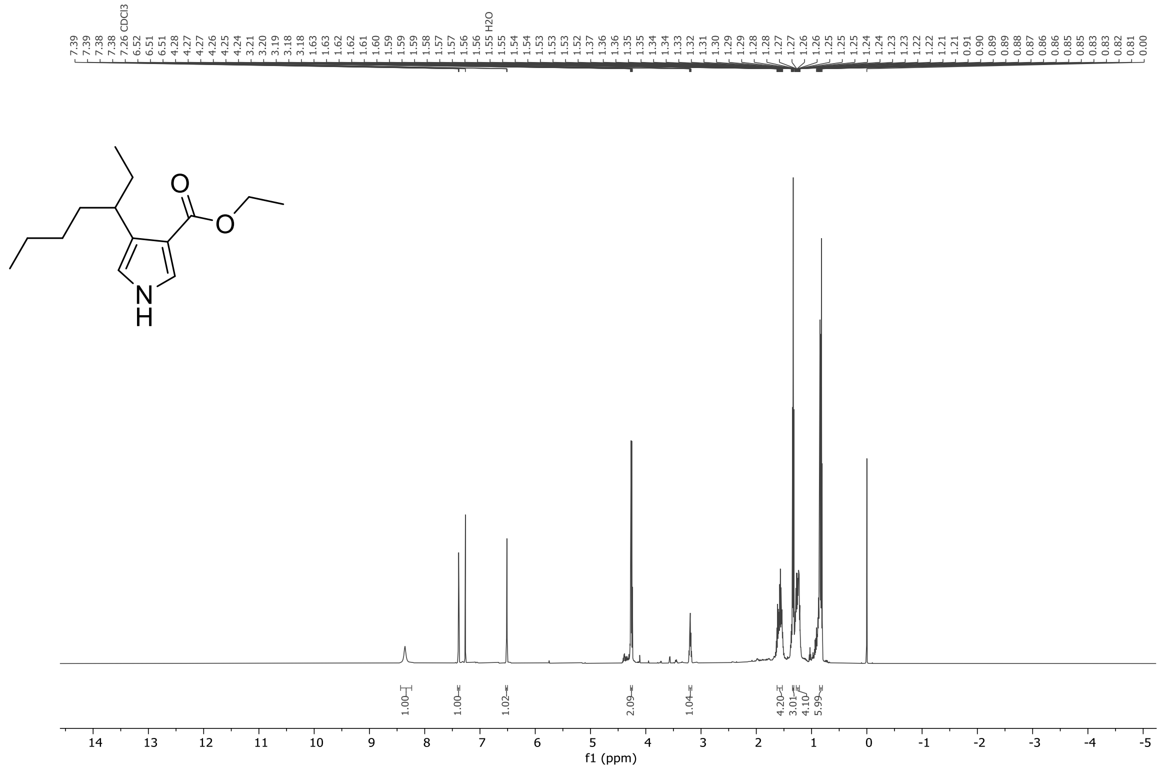

Figure S25. ^{1}H-NMR (600 MHz, $CDCl_3$) spectrum of **2.**

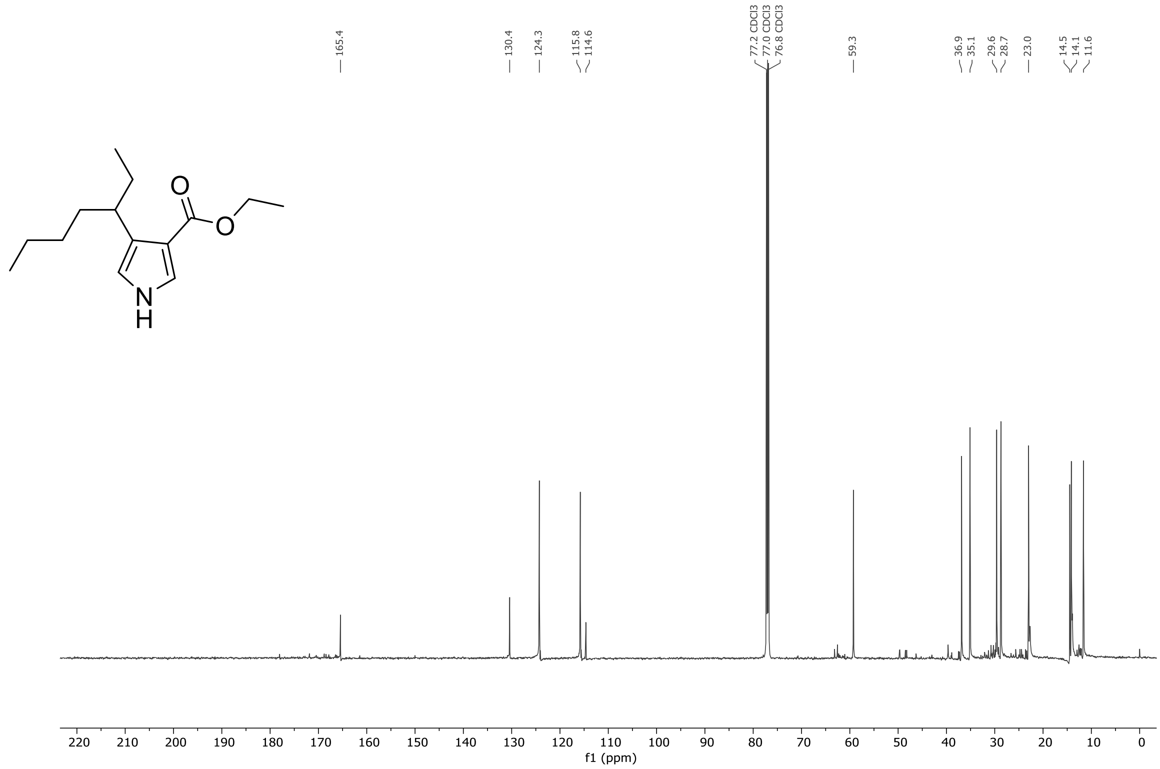

Figure S26. ^{13}C-NMR (600 MHz, $CDCl_3$) spectrum of **2.**

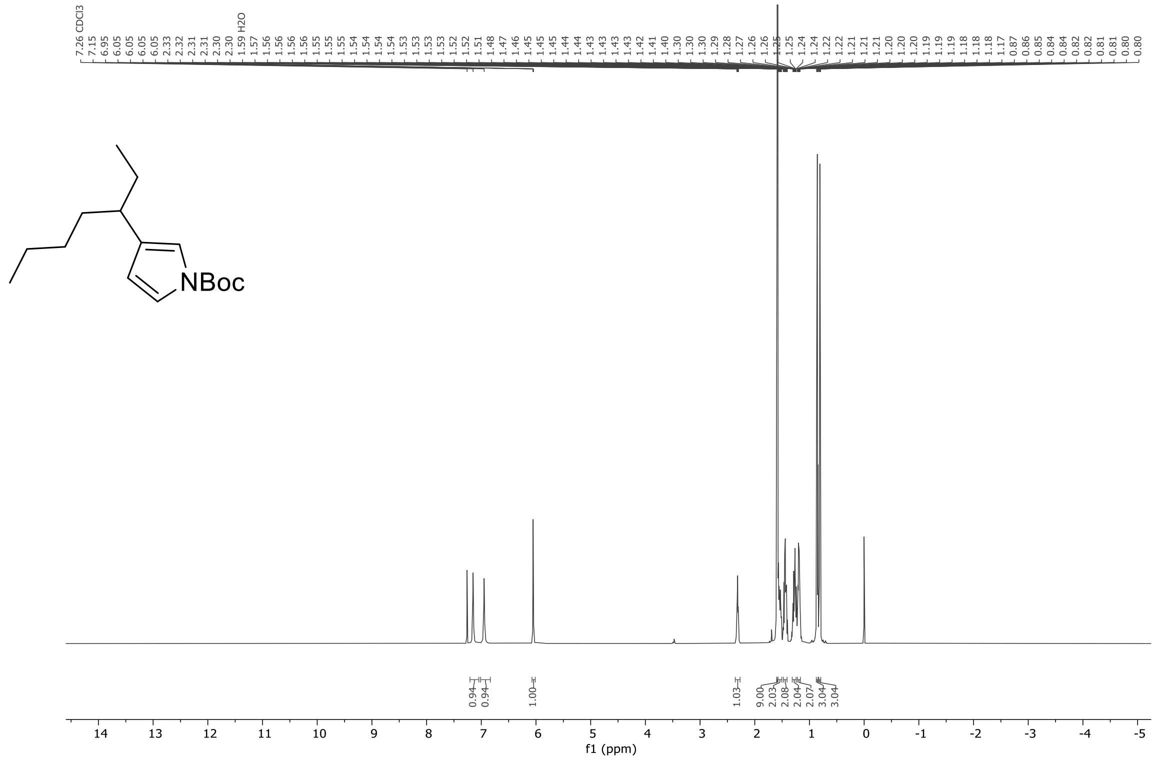

Figure S27. ^{1}H-NMR (600 MHz, $CDCl_3$) spectrum of **3.**

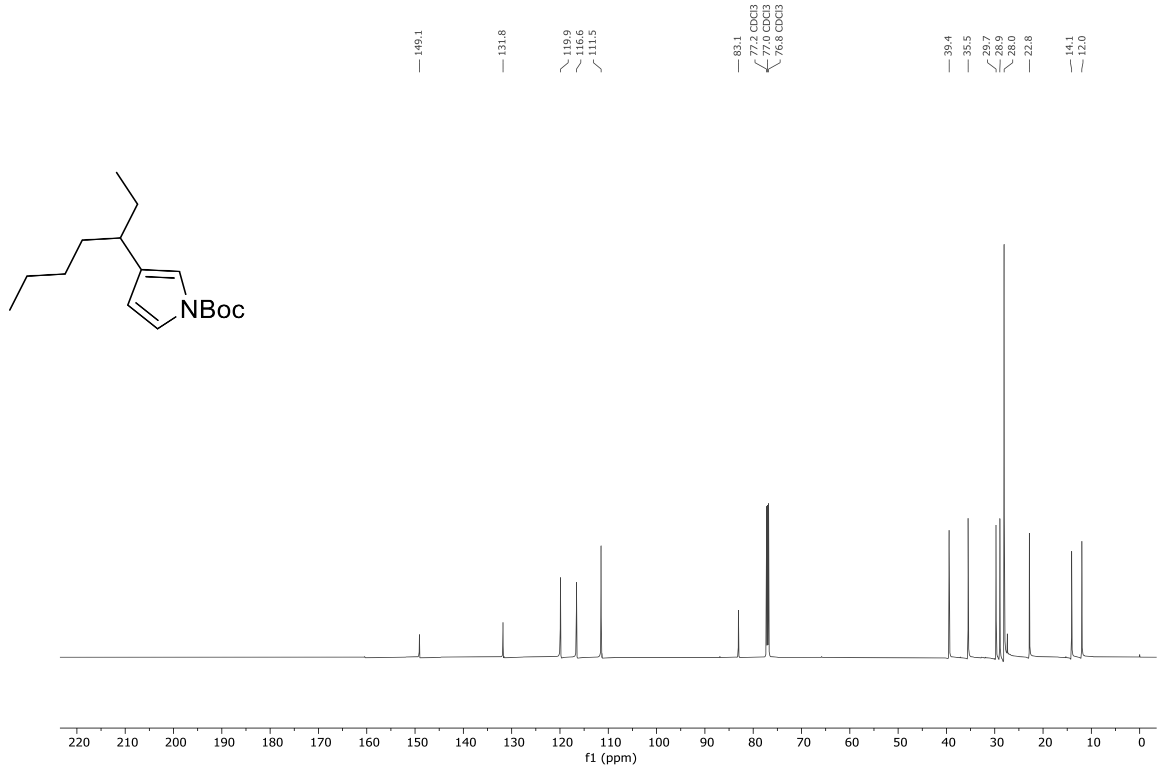

Figure S28. ^{13}C-NMR (600 MHz, $CDCl_3$) spectrum of **3.**

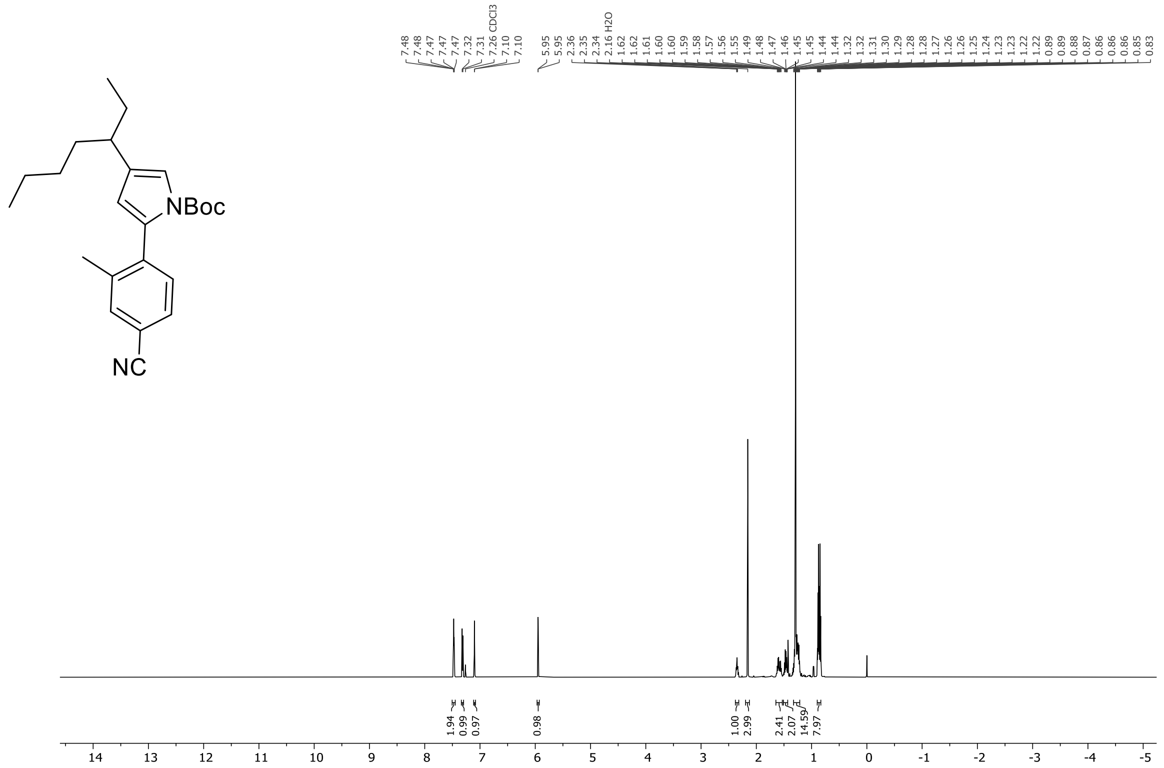

Figure S29. ^{1}H-NMR (600 MHz, $CDCl_3$) spectrum of **4.**

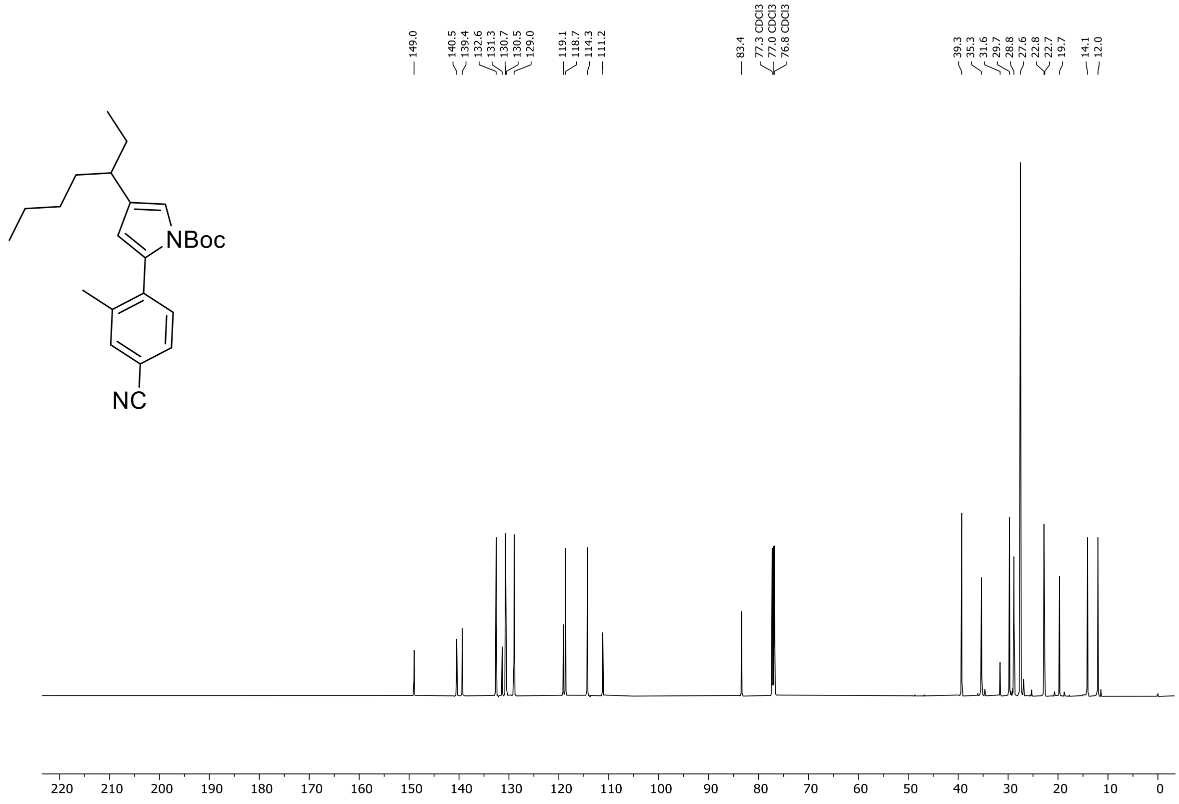

Figure S30. ^{13}C-NMR (600 MHz, $CDCl_3$) spectrum of **4.**

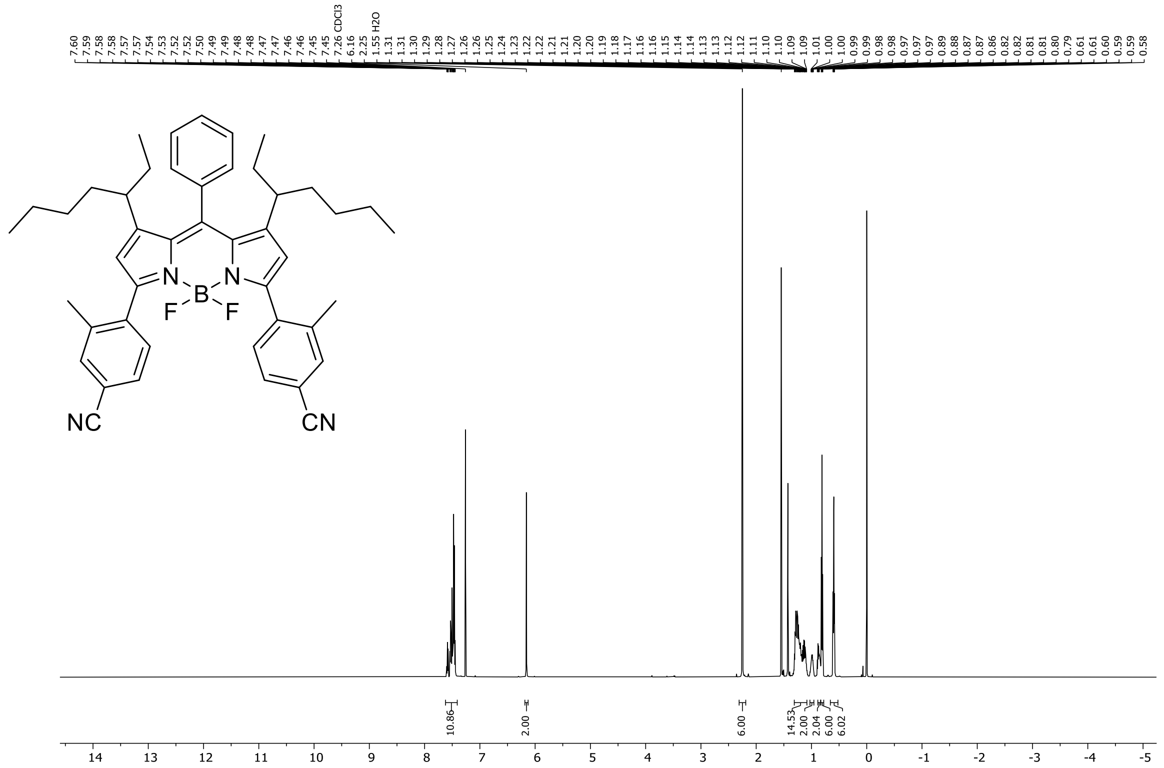

Figure S31. ^{1}H-NMR (600 MHz, $CDCl_3$) spectrum of the **BODIPY derivative.**

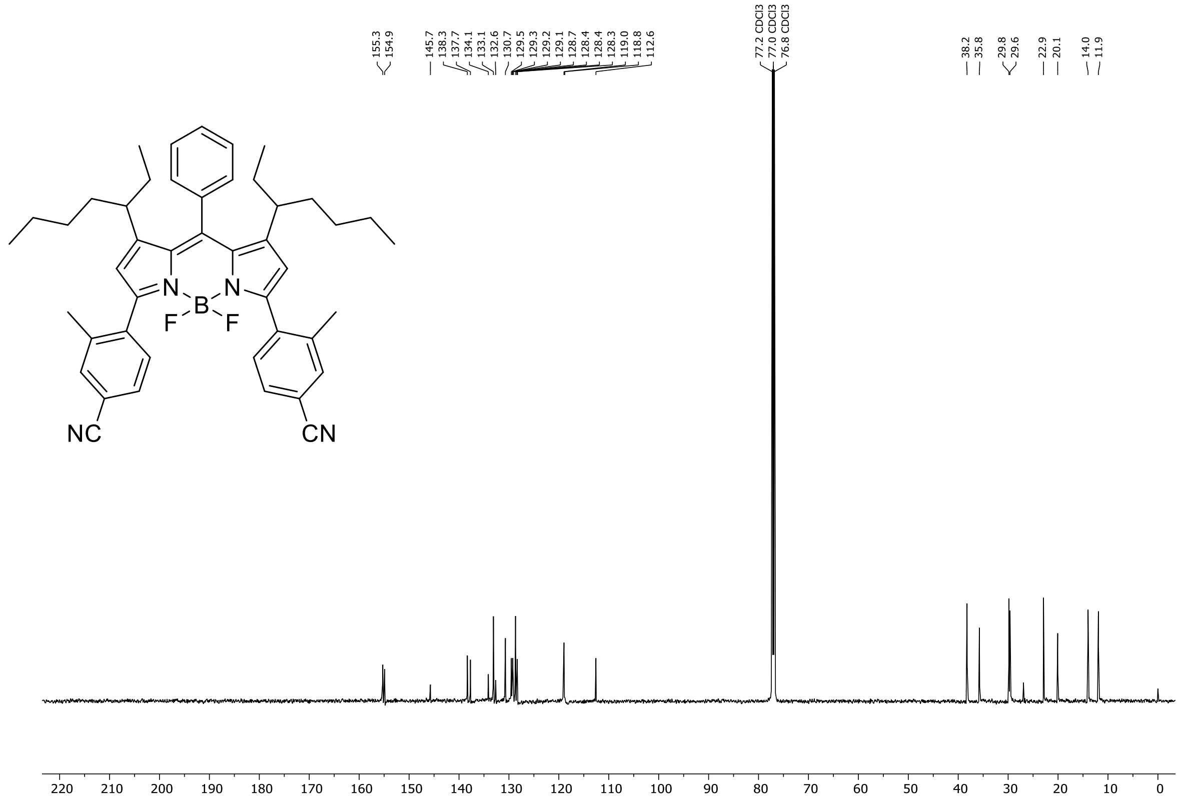

Figure S32. ^{13}C-NMR (600 MHz, $CDCl_3$) spectrum of the **BODIPY derivative.**

.

6. Supplementary Tables

Table-S1: Photophysical properties of the BODIPY derivative. Where ε is the absorptivity coefficient, Φ is the emission quantum yield, and τ_1 and τ_2 is the excited state lifetime.

	Toluene solution	Neat film
ε ($M^{-1}cm^{-1}$)	77600 (512nm)	
Φ	0.37[a]	0.33±0.02[b]
τ_1 (ns)	1.94	1.405(0.95)[c]
τ_2 (ns)		2.748 (0.05)[c]

[a]Fluorescein in 0.1 M NaOH (Φ = 0.91) was used as a reference compound for the Φ determination. (Excitation at 491 nm, Refractive index: 1.33). [b]Quantum yields of the film was measured using an integrating sphere. [c]Pre-exponential factor in parentheses.

Table-S2: Values extracted from the coupled harmonic oscillator model, where the excitonic energy was set to E_x = 2.43 eV. Here, n_{eff} represents the refractive index, $E_c(0)$ represents the cavity energy at $k_{\parallel} = 0$, and V_a represents the coupling strength.

Cavity	E_X (eV)	E_c (0) (eV)	n_{eff}	V_a (meV)	L_{cav} (nm)
Cav-1-TE	2.43	2.277	1.932	319	141
Cav-2-TE	2.43	2.395	1.932	310	134
Cav-3-TE	2.43	2.529	1.932	306	127
Cav-1-TM	2.43	2.277	3.354	321	81
Cav-2-TM	2.43	2.395	3.160	310	82
Cav-3-TM	2.43	2.529	3.072	304	80

Table-S3: The source terms coefficients for different dipoles.

	TE	TM
Horizontal dipoles	$A_t, A_b = \pm\sqrt{\frac{3}{8\pi}}$	$A_t, A_b = \sqrt{\frac{3}{8\pi}}\cos(\theta_{mol})$
Vertical dipoles	0	$A_t, A_b = \pm\sqrt{\frac{3}{8\pi}}\sin(\theta_{mol})$

7. Reference

(1) Schäfer, C.; Hultmark, S.; Yang, Y. Z.; Muller, C.; Börjesson, K. Room Temperature Dye Glasses: A Guideline Toward the Fabrication of Amorphous Dye Films with Monomeric Absorption and Emission. *Chem Mater* **2022**, *34* (20), 9294-9302.
(2) Chen, S. L.; Fu, L.; Gan, W.; Wang, H. F. Homogeneous and inhomogeneous broadenings and the Voigt line shapes in the phase-resolved and intensity sum-frequency generation vibrational spectroscopy. *J. Chem. Phys.* **2016**, *144* (3), 034704.
(3) Al-Ghamdi, M. S.; Bahnam, R. Z.; Karomi, I. B. Study and analysis of the optical absorption cross section and energy states broadenings in quantum dot lasers. *Heliyon* **2022**, *8* (9), e10587.
(4) M.J., W. *Handbook of Optical Materials*; CRC Press, 2002.
(5) Benisty, H.; Stanley, R.; Mayer, M. Method of source terms for dipole emission modification in modes of arbitrary planar structures. *J. Opt. Soc. Am. A.* **1998**, *15* (5), 1192-1201.
(6) Lukosz, W. Light-Emission by Magnetic and Electric Dipoles Close to a Plane Dielectric Interface .3. Radiation-Patterns of Dipoles with Arbitrary Orientation. *J. Opt. Soc. Am.* **1979**, *69* (11), 1495-1503.
(7) Lukosz, W.; Kunz, R. E. Light-Emission by Magnetic and Electric Dipoles Close to a Plane Dielectric Interface .2. Radiation-Patterns of Perpendicular Oriented Dipoles. *J. Opt. Soc. Am.* **1977**, *67* (12), 1615-1619.
